# Towards the description of charge transfer states in solubilised LHCII using subsystem DFT

**DOI:** 10.1007/s11120-022-00950-7

**Published:** 2022-08-21

**Authors:** Souloke Sen, Lucas Visscher

**Affiliations:** Division of Theoretical Chemistry, Faculty of Sciences, Vrije Universiteit Amsterdam, De Boelelaan 1083, 1081 HV Amsterdam, The Netherlands

**Keywords:** Charge transfer, Exciton, Chromophore, Diabatic

## Abstract

Light harvesting complex II (LHCII) in plants and green algae have been shown to adapt their absorption properties, depending on the concentration of sunlight, switching between a light harvesting and a non-harvesting or quenched state. In a recent work, combining classical molecular dynamics (MD) simulations with quantum chemical calculations (Liguori et al. in Sci Rep 5:15661, 2015) on LHCII, it was shown that the Chl611–Chl612 cluster of the terminal emitter domain can play an important role in modifying the spectral properties of the complex. In that work the importance of charge transfer (CT) effects was highlighted, in re-shaping the absorption intensity of the chlorophyll dimer. Here in this work, we investigate the combined effect of the local excited (LE) and CT states in shaping the energy landscape of the chlorophyll dimer. Using subsystem Density Functional Theory over the classical $\mu$*s* MD trajectory we look explicitly into the excitation energies of the LE and the CT states of the dimer and their corresponding couplings. Upon doing so, we observe a drop in the excitation energies of the CT states, accompanied by an increase in the couplings between the LE/LE and the LE/CT states facilitated by a shorter interchromophoric distance upon equilibration. Both these changes in conjunction, effectively produces a red-shift of the low-lying mixed exciton/CT states of the supramolecular chromophore pair.

**Supplementary Information:**

The online version contains supplementary material available at 10.1007/s11120-022-00950-7.

## Introduction

Light harvesting complex II (LHCII) or the major antenna complex of the Photosystem-II (PSII) in higher plants and green algae, is a complex structure accounting for almost 80% of the light absorbed by PSII. LHCII exists in the native trimeric form where one monomer consists of eight chlorophyll *a* (Chla), six chloropyll *b* (Chlb), two luteins (Lut), one neoxanthin (Nx) and one violaxanthin (Vx) chromophore as cofactors. The trimeric (major) LHCII and the monomeric (minor) LHCII complexes (CP24, CP26 and CP29) along with the PSII core form the PSII supercomplex, the crystal structure of which has been determined at high resolution (Wei et al. 2016; Su et al. 2017). Previous studies on LHCs, have highlighted the importance of the excitation energy transfer (EET) processes in fine tuning the spectral properties of the complexes (Segatta et al. 2019; Cupellini et al. 2020b; Balevičius et al. 2017; Maity et al. 2019; Duffy et al. 2013; Chmeliov et al. 2015; Lapillo et al. 2020; Khokhlov and Belov 2019). [We refer the reader to reference (Segatta et al. 2019) for a focused review on the different theoretical methods and to reference (Cupellini et al. 2020b) for the challenges therein]. In addition Electron Transfer (ET) effects, giving rise to Charge Transfer (CT) states, have also been shown to play a crucial role in modulating the overall spectral and energy landscape of the complexes especially for closely interacting pigments (Kell et al. 2014; Wahadoszamen et al. 2014; Novoderezhkin et al. 2007; Miloslavina et al. 2008; Cupellini et al. 2020a). Although CT states by themselves are optically dark states, previous theoretical studies have shown that these states can mix with the local states thereby gaining some dipole strength (Cupellini et al. 2018; Nottoli et al. 2018). Previous studies have also elucidated a strong correlation between the LHC energetics and protein conformational changes (Ruban et al. 2012; Krüger 2014; Liguori et al. 2013), showing that the so-called ‘quenched’ states of LHCs are linked to particular conformations where the chromophore–chromophore interactions are strengthened (Pascal et al. 2005; Ruban et al. 2007; Ahn et al. 2008; Bode et al. 2009; Miloslavina et al. 2008). In this respect, interactions between a particular chlorophyll dimer (originally labelled Chla611–Chla612 in reference Liu et al. 2004) is particularly interesting because it is often assumed to be a critical site for the switch between light harvesting and quenched conformations of LHCs (Ruban et al. 2007; Mozzo et al. 2008; Mascoli et al. 2019).

The advent of Classical Molecular Dynamics (MD) simulations in the microsecond ($\mu s$) time scale allows for probing the chromophore dynamics at atomistic resolution, also allowing the inclusion of lipid membrane and solvent effects (Curutchet and Mennucci 2017). Snapshots from these long simulations can be used as input for quantum chemical calculations to provide a computationally efficient strategy for studying spectral properties of LHCs over such time scales. We note that care has to be taken that the force field reproduces the structure of the chromophores well enough (Andreussi et al. 2017; Jurinovich et al. 2015) and have checked in earlier work (Sen et al. 2021) that the difference of excitation energies between MM and QM optimized structures does not exceed 0.1 eV. In that work, we performed Time-Dependent Density Functional Theory (TDDFT) (Runge and Gross 1984; Casida 1995) calculations on the above chlorophyll dimer (i.e. Chla611–Chla612) on selected snapshots from a particular trajectory obtained from a $\mu s$ classical MD simulation which showed significant structural disorder from the crystal structure (Liguori et al. 2015). This particular trajectory [trajectory “A” in the original work of reference (Liguori et al. 2015) that was also used in reference (López-Tarifa et al. 2017)] was one of six independent simulations where the excitonic interactions between the chlorophyll dimer displayed marked differences compared to the others (Liguori et al. 2015). In our previous work, it was shown that a drop in the total oscillator strength of the *Q* bands ($Q_{y}$ and $Q_{x}$) could be explained using a transition density decomposition analysis, wherein an increase in the CT component of the oscillator strength was observed upon equilibration. Furthermore a characterization of the excitonic states using charge transfer descriptors of Plasser and Lischka (2012) revealed an overall increase of the CT character of the low-lying states. Both of these analysis clearly showed an increase in the mixing of the CT states with the local excited (LE) *Q* states, making it difficult to make a quantitative calculation of the energies of the LE and the CT states towards the end of the trajectory.

**Fig. 1:**
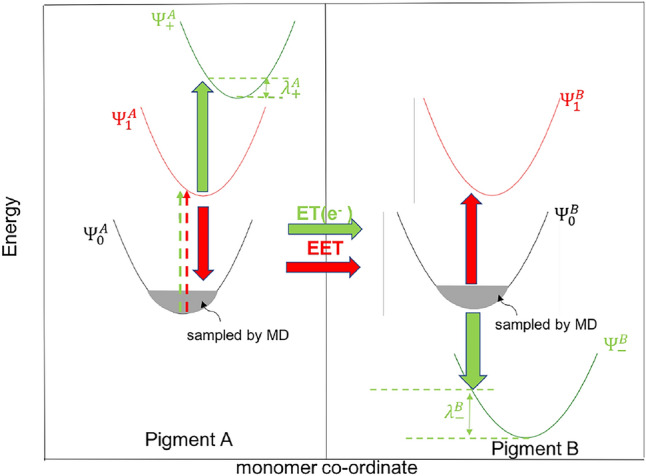
Excitation energy transfer and electron transfer are represented in the monomer coordinate system. The red arrows represents Förster (Förster 1948) energy transfer flow, whereas the green arrows represent the Marcus (Marcus and Sutin 1985) electron transfer between pigments *A* and *B*. Vertical excitation processes are represented with broken arrows. The ground, local excited and ionized potential surfaces of the corresponding pigments *A* and *B* are shown in black, red and green (denoted by $\Psi ^{A/B}_{i}$) respectively in the increasing order of energy. $\lambda ^{A/B}_{i}$ denotes the reorganization energy associated with the electronic transition

In this previous work, the delocalized orbital picture resulting from the use of a supramolecular basis made the interpretation of the TDDFT states difficult when the distance between the chromophores was small. In the current work, we instead use a diabatic representation built on the framework of subsystem DFT in which the identification as LE or CT states becomes straightforward. A pictorial description of the involved electronic states for both these processes for two identical pigments is shown in Fig. 1. We construct a model Hamiltonian that can be used to predict the importance of the ET and EET processes by computing the subsystem DFT energies of the individual diabatic states (diagonal elements of the model Hamiltonian) as well as their couplings (the off-diagonal elements). We thereby focus on the pair Chla611–Chla612 and compare computed matrix elements from the beginning and the end of the MD trajectory. Upon doing so, we observe a decrease in the energies of the CT states which are accompanied by an increase in the coupling between the LE and CT states and among the LE states themselves, resulting from a shorter interchromophoric distance between Chla611 and Chla612 after equilibration. We show that this conformational change results in a red-shift of the low-lying mixed excitonic/CT state, induced by a drop in the CT state energy and an increased coupling between the local and CT state and between the local states themselves.

This paper is organized as follows: In the “Methods” section, we introduce the model Hamiltonian and give an overview of the subsystem DFT framework. Therein, the defining equations for the approximations used in the calculation of the matrix elements of the effective Hamiltonian (representing the local and CT energies and their couplings) are discussed. In the “Computational details” section, the technical details of the workflow and the calculations performed to obtain the matrix elements are specified. In the “Results and discussion” section, we then discuss the obtained values of the matrix elements of the model Hamiltonian and compare with the supramolecular picture. In the final concluding section we also present a brief outlook for further studies.

## Methods

In order to obtain approximate solutions of the Schrödingers equation of a many-body system involving electrons and nuclei one usually employs the Born–Oppenheimer approximation (1927). In this approximation the total wavefunction of the system, $\Psi _{i\mu }(\mathbf {R},\mathbf {r})$, where $\mathbf {R}$ and $\mathbf {r}$ represent the set of coordinates of the nuclei and the electrons, respectively, is written as a product of the electron and nuclear wavefunctions, i.e.

1 $$\begin{aligned} \Psi _{i\mu }(\mathbf {R},\mathbf {r}) := \Phi _{i}(\mathbf {R},\mathbf {r}) \chi _{i\mu }(\mathbf {R}) \end{aligned}$$

where $\Phi _{i}$ is $i{\text{th}}$ electronic wave function obtained for a fixed nuclear configuration $\mathbf {R}$, and the $\chi _{i\mu }$ are nuclear wave functions for vibrational states $\mu$. The product of these two wave functions, $\Phi _{i}(\mathbf {R},\mathbf {r}) \chi _{i\mu }(\mathbf {R})$, describes so-called vibronic states $i\mu$ and their energy, $E_{i\mu }$. Vibronic transitions are associated with a change in both the electronic and the vibrational states, the corresponding rate of which is given by the Fermi’s golden rule,

2 $$\begin{aligned}&k_{i\mu , j\nu } = \frac{2\pi }{\hbar } \vert H^1_{i\mu j\nu }\vert ^{2} \delta (E_{i\mu } - E_{j\nu }) , \end{aligned}$$

3 $$\begin{aligned}&H^1_{i\mu , j\nu } = \langle \chi _{i\mu }(\mathbf {R})\vert \langle \Phi _{i}(\mathbf {R},\mathbf {r})\vert {\hat{H}}^1\vert \Phi _{j}(\mathbf {R},\mathbf {r})\rangle \vert \chi _{j\nu }(\mathbf {R}) \rangle \end{aligned},$$

where $k_{i\mu j\nu }$ is the rate, ${\hat{H}}^1$ a perturbation operator and $E_{i\mu }$, $E_{j\nu }$ are the energies of the initial and final vibronic states $i\mu$ and $j\nu$ respectively. In the Franck–Condon approximation, the matrix element, $H^1_{i\mu j\nu }$ is separated into two factors, i.e.,

4 $$\begin{aligned} H^1_{i\mu , j\nu } = \langle \Phi _{i}(\mathbf {R},\mathbf {r})\vert {\hat{H}}^1\vert \Phi _{j}(\mathbf {R},\mathbf {r})\rangle \, . \, \langle \chi _{i\mu }(\mathbf {R})\vert \chi _{j\nu }(\mathbf {R}) \rangle \end{aligned}$$

The first term on the rhs of Eq. 4 represents the coupling matrix element between the electronic states *i* and *j* that is our main interest. The nuclear wavefunction overlap matrix elements, given by the second term on the rhs of Eq. 4, determine the homogenous (vibrational) broadening of the transition between the two electronic states *i* and *j* and will not be considered explicitly.

**Fig. 2:**
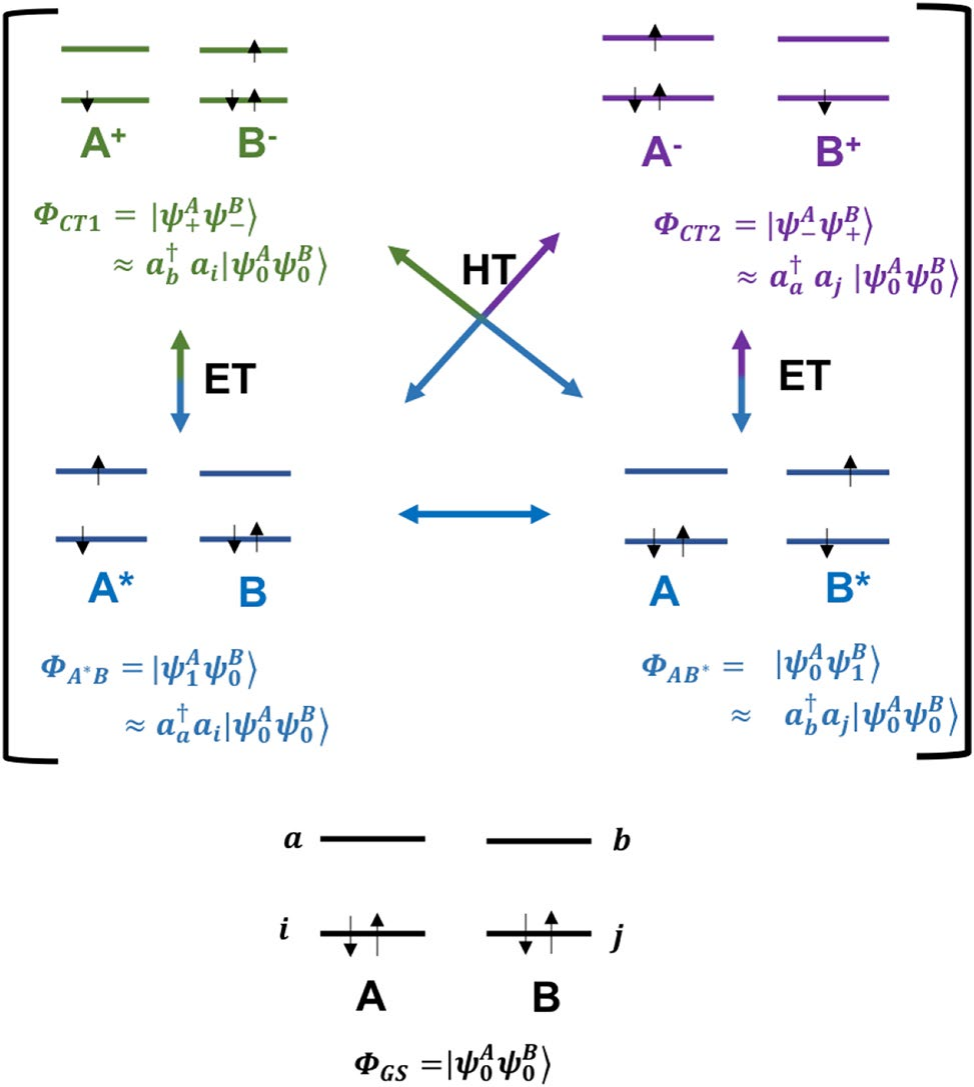
The ground state, $\Phi _{\text{GS}}$, two locally excited states, $\Phi _{A^{*}B}$ and $\Phi _{AB^{*}}$, and two CT states, $\Phi _{\text{CT1}}$ , $\Phi _{\text{CT2}}$ along with the distribution of electrons, where each fragment is considered as a two-level two electron system, are shown. $a^{\dagger }$ and *a* are the corresponding creation and annihilation operators, and *i*, *j* and *a*, *b* represent occupied and unoccupied orbitals of the fragments *A* and *B*, respectively

We aim to calculate the matrix elements of the electronic Hamiltonian $\hat{H}^{\text{el}}$ in a diabatic basis, consisting of the electronic ground state $\Phi _{\text{GS}}$ and sets of excited states $\{\Phi _{A^{*}B}\}$, $\{\Phi _{AB^{*}}\}$, $\{\Phi _{\text{CT1}}\}$ and $\{\Phi _{\text{CT2}}\}$, where states $\Phi _{A^{*}B}$, $\Phi _{AB^{*}}$ describe LE states of chromophores *A* and *B*, and $\Phi _{\text{CT1}}$, $\Phi _{\text{CT2}}$ describe the non-local charge transfer states ($A^{+}B^{-}$ and $A^{-}B^{+}$) respectively. This basis is illustrated graphically in Fig. 2. In the subsystem, or weak coupling, approach, these states can be written as a product of local states,

5 $$\begin{aligned} \Phi _{\text{GS}}= & {} \vert \psi ^{A}_{0}\psi ^{B}_{0}\rangle \end{aligned}$$

6 $$\begin{aligned} \Phi _{A^{*}B}= & {} \vert \psi ^{A}_{1}\psi ^{B}_{0}\rangle \end{aligned}$$

7 $$\begin{aligned} \Phi _{AB^{*}}= & {} \vert \psi ^{A}_{0}\psi ^{B}_{1}\rangle \end{aligned}$$

8 $$\begin{aligned} \Phi _{\text{CT1}}= & {} \vert \psi ^{A}_{+}\psi ^{B}_{-}\rangle \end{aligned}$$

9 $$\begin{aligned} \Phi _{\text{CT2}}= & {} \vert \psi ^{A}_{-}\psi ^{B}_{+}\rangle \end{aligned},$$

where $\psi _{0}^{A/B}$, $\psi _{1}^{A/B}$, $\psi _{+}^{A/B}$ and $\psi _{-}^{A/B}$ are, respectively, the ground state, LE state, ionized and electron-attached wavefunctions for subsystems *A* or *B*. Note that we have dropped the explicit coordinate dependence of the set of $\Phi _{i}$’s on $\mathbf {R}$ and $\mathbf {r}$ in the above definitions for convenience. In this diabatic basis the Hamiltonian matrix assumes a blocked structure with diagonal blocks representing the electronic ground state ($\mathbf {H}^{\text{el}}_{\text{GS}}$), interactions between LE states, ($\mathbf {H}^{\text{el}}_{\text{LE}}$), as well as interactions between CT states ($\mathbf {H}^{\text{el}}_{\text{CT}}$). Off-diagonal blocks ($\mathbf {H}^{\text{el}}_{X-Y}$, where $X,Y \in (\text{GS, LE, CT})$ and $X \ne Y$) couple these physically distinct states and lead to states with mixed LE–CT character.

In order to describe the final states we need to compute the values of the matrix elements in each of the blocks. We can set the energy of the ground state to zero and define the diagonal matrix elements of $\mathbf {H}^{\text{el}}_{\text{LE}}$ and $\mathbf {H}^{\text{el}}_{\text{CT}}$ block, i.e. $\langle \Phi _{i}\vert {\hat{H}}^{\text{el}}\vert \Phi _{i}\rangle$, where, $i \in (A^{*}B, AB^{*}, \text{CT1}, \text{CT2})$ as LE and CT excitation energies. The calculation of off-diagonal matrix elements inside the $\mathbf {H}^{\text{el}}_{\text{LE}}$ block, i.e. $\langle \Phi _{i}\vert {\hat{H}}^{\text{el}}\vert \Phi _{j}\rangle,$ where $i,j \in (A^{*}B,AB^{*})$ and $i \ne j$ is facilitated by the fact that the two transition densities are (almost) non-overlapping. We furthermore note that off-diagonal elements in $\mathbf {H}^{\text{el}}_{\text{CT}}$ block, i.e. the coupling between CT states in which the transfer goes in opposite directions can be assumed negligible as they correspond to a two-electron process. The couplings between the GS and CT state and between the GS and LE states are accounted for in the calculation of the CT and LE energies and need not be considered explicitly. The final significant coupling is between LE and CT states and will be calculated in a generalization of Fragment Orbital DFT (Senthilkumar et al. 2003, 2005) that we describe below.

**Fig. 3:**
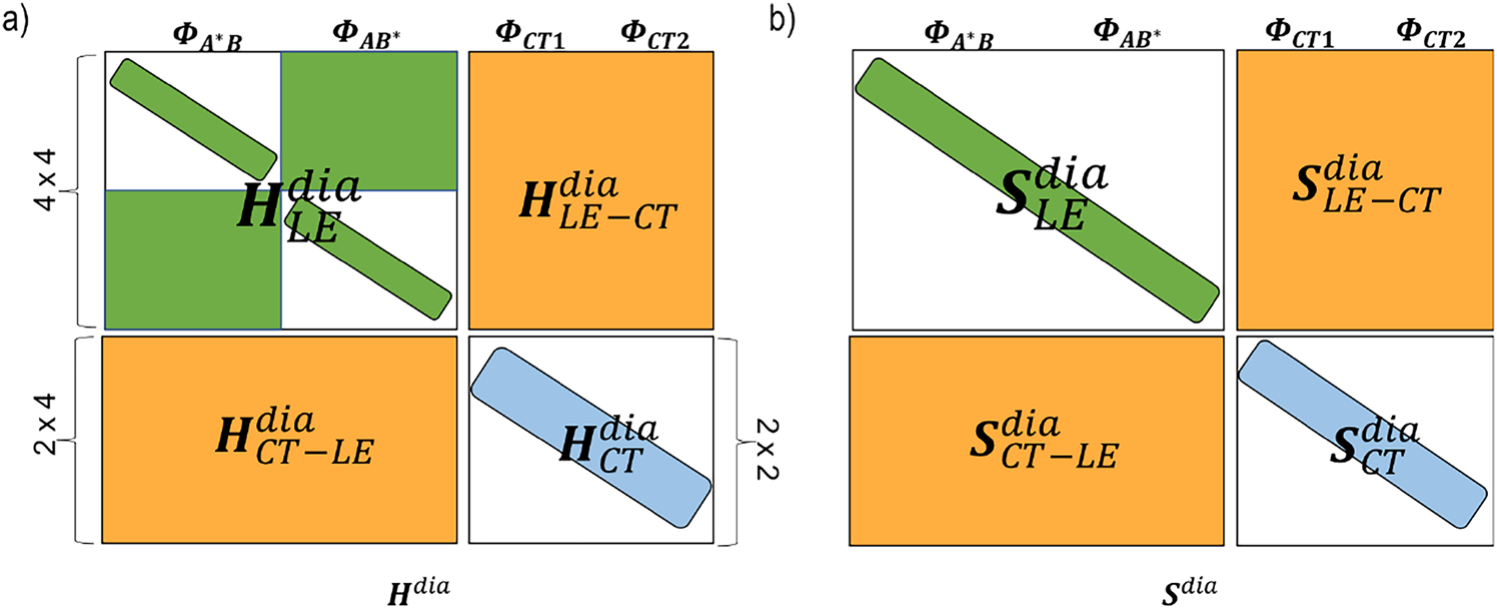
Structure of **a** the Hamiltonian matrix, $\mathbf {H}^{\text{dia}}$, and **b** the Overlap matrix, $\mathbf {S}^{\text{dia}}$ which is used in this work. LE represents the locally excited states on pigment *A* and *B*, and CT the, non-local charge transfer states. The $\mathbf {H}^{\text{dia}}_{\text{LE}}$, $\mathbf {H}^{\text{dia}}_{\text{CT}}$ and $\mathbf {H}^{\text{dia}}_{{\text{LE}}{-}{\text{CT}}}$ blocks, constructed following Table 1 are shown in green, blue and orange, respectively. We assume zero coupling (white areas) between the CT states of different polarity

As the diabatic states will be constructed independently from each other, they are in general non-orthogonal and adiabatic eigen states $\Psi ^{\text{ad}}$ and energies $E^{\text{ad}}$ need therefore be determined by solving a generalized eigenvalue problem

10 $$\begin{aligned} \mathbf {H}^{\text{dia}} \mathbf {C}^{\text{ad}} = E^{\text{ad}} \mathbf {S}^{\text{dia}}\mathbf {C}^{\text{ad}} \end{aligned},$$

where $\mathbf {H}^{\text{dia}}$ denotes the diabatic matrix representation of $\mathbf {H}^{\text{el}}$. The overlap matrix $\mathbf {S}^{\text{dia}}$ is constructed in addition to evaluation of the matrix elements of $\mathbf {H}^{\text{dia}}$ and $\mathbf {C}^{\text{ad}}$ are the coefficients of the adiabatic eigenstates in the diabatic basis. As the coupling between the ground state and the locally excited states is zero by construction, and the coupling between the ground state and the charge transfer states is very weak for the systems we are considering, we thereby focus only on the set of diabatic states {$\Phi _{A^{*}B}$, $\Phi _{AB^{*}}$, $\Phi _{\text{CT1}}$ $\Phi _{\text{CT2}}$}. The structure of the matrices $\mathbf {H}^{\text{dia}}$ and $\mathbf {S}^{\text{dia}}$ used in this work is shown in Fig. 3. We note that $\mathbf {H}^{\text{dia}}$ shares a similar structure with other ab-initio exciton models used to describe such processes (Cupellini et al. 2018; Nottoli et al. 2018). In the next sections we will introduce a composite approach to obtain the non-zero matrix elements.

**Table 1:** Summary of the equations/approximations used to evaluate the different blocks of $\mathbf {H}^{\text{dia}}$

Blocks		Equations	Description/References
$\mathbf {H}^{\text{dia}}_{\text{LE}}$	$\langle \Phi _{\text{sub}}\vert {\hat{H}}^{\text{el}}\vert \Phi _{\text{sub}}\rangle$	$$\left( \begin{array}{cc} {\textbf {A}}^{\text{sub}} &{} {\textbf {B}}^{\text{sub}} \\ -{\textbf {B}}^{\text{sub}} &{} -{\textbf {A}}^{\text{sub}} \end{array}\right) \left( \begin{array}{c} {\textbf {X}}^{\text{sub}} \\ {\textbf {Y}}^{\text{sub}} \end{array}\right) = \mathbf {\omega } \left( \begin{array}{cc} {\textbf {X}}^{\text{sub}} \\ {\textbf {Y}}^{\text{sub}} \end{array}\right)$$ (Eq. 21)	$\text{sub} \in \big \{A^{*}B, AB^{*}\big \}$; FDEu-TDDFT; Refs. Neugebauer (2007), König et al. (2013)
	$\langle \Phi ^\mu _{A^{*}B}\vert {\hat{H}}^{\text{el}}\vert \Phi ^\nu _{AB^{*}}\rangle$	$\int \int \rho _{A,\mu }(\mathbf {r})f(\mathbf {r}, \mathbf {r'})\rho _{B,\nu }(\mathbf {r'})\text{d}\mathbf {r}\text{d}\mathbf {r'}$ (Eq. 22)	FDEc-TDA; Ref. König et al. (2013)
$\mathbf {H}^{\text{dia}}_{\text{CT}}$	$\langle \Phi _{X}\vert {\hat{H}}^{\text{el}}\vert \Phi _{X}\rangle$	$H^{\text{dia}}_{XX} - H^{\text{dia}}_{00}$ (Eq. 27)	$X \in \big \{\text{CT1},\text{CT2}\big \}$; FDE-ET; Refs. Pavanello and Neugebauer (2011), Pavanello et al. (2013), Solovyeva et al. (2014), Ramos et al. (2015)
$\mathbf {H}^{\text{dia}}_{{\text{LE}}{-}{\text{CT}}}$	$\langle \Phi _{A^{*}B}\vert {\hat{H}}^{\text{el}}\vert \Phi _{\text{CT1}}\rangle$	$\sum _{a} \varepsilon _a \delta _{\kappa \lambda }S'_{a\alpha _A}S'_{a\beta _B}$ $-\sum _{i} \varepsilon _i S_{\alpha _A,\beta _B}S'_{i,\kappa _A}S'_{i,\lambda _A}$ (Eq. 36)	Generalized FODFT; Refs. Senthilkumar et al. (2003, 2005); see SI for remaining couplings
	$\langle \Phi _{A^{*}B}\vert {\hat{H}}^{\text{el}}\vert \Phi _{\text{CT2}}\rangle$	$-\sum _{i} \varepsilon _i \delta _{\alpha \beta }S'_{i\kappa _A}S'_{i\lambda _B}$ $+\sum _{a} \varepsilon _a S_{\kappa _A,\lambda _B}S'_{a,\alpha _A}S'_{a,\beta _A}$ (Eq. 41)	

### Subsystem DFT

While the theory has thus far been general and applicable with any diabatic wave function ansatz, we will now specialize to the wave functions provided by subsystem DFT (Jacob and Neugebauer 2014; Wesolowski et al. 2015). Subsystem DFT is typically formulated such that a monomer basis set is used to represent a subsystem and will thereby by construction yield local states. We, therefore construct each of the aforementioned diabatic states from separate sets of (spin)orbitals of *A* and *B* resulting from an underlying subsystem DFT calculation. The ground state determinant, $\Phi _{\text{GS}}$ in Eq. 5 (Fig. 2) of the combined system *A* + *B* in the subsystem framework is thereby written as combination of two sets of subsystem spinorbitals $\{\phi _{A}^{i}\}$ and $\{\phi _{B}^{j}\}$ on *A* and *B* comprising $\psi ^{A}_{0}$ and $\psi ^{B}_{0}$ in Eq. 5 i.e,

11 $$\begin{aligned} \Phi _{\text{GS}} = \vert \psi ^{A}_{0}\psi ^{B}_{0}\rangle \approx \frac{1}{\sqrt{N_{A}+N_{B}}}\bigg \Vert \phi _{A}^{1}...\phi _{A}^{i}...\phi _{A}^{N_{A}}\phi _{B}^{1}...\phi _{B}^{j}..\phi _{B}^{N_{B}}\bigg \Vert \end{aligned}$$

In the following section we briefly outline the basic formulation of subsystem DFT and thereafter introduce the different flavours that are used in approximating the matrix elements of $\mathbf {H}^{\text{dia}}$.

The basic idea of subsystem DFT is to partition the electron density of a total system into subsystem contributions (Wesolowski and Warshel 1993)

12 $$\begin{aligned} \rho (\mathbf {r}) = \sum _{K}^{n} \rho _{K}(\mathbf {r}) \end{aligned},$$

where n is the number of subsystems. The total energy of the system is then minimized with respect to all $\rho _{K}$, where each of the subsystem densities is usually represented by a Kohn–Sham like system of independent particles. In case of two subsystems *A* and *B*, the ground state energy of *A* + *B* is then given by,

13 $$\begin{aligned} E_{0}= & {} \min _{\rho _{{A}},\rho _{{B}}} \{ T_{s}[\rho _{{A}}] + T_{s}[\rho _{{B}}] + T_{s}^{\text{nad}}[\rho _{{A}}, \rho _{{B}}] + V_{\text{nuc}}[\rho _{{A}} + \rho _{{B}}]\nonumber \\&+ J[\rho _{{A}} + \rho _{{B}}] + E_{\text{xc}}[\rho _{{A}} + \rho _{{B}}] \}, \end{aligned}$$

where

14 $$\begin{aligned} T_{s}^{\text{nad}}[\rho _{{A}} , \rho _{{B}}] = T_{s}[\rho _{{A}} + \rho _{{B}}] - T_{s}[\rho _{{A}}] - T_{s}[\rho _{{B}}] \end{aligned}$$

Therefore in subsystem DFT, in addition to the usual $E_{\text{xc}}$ functional, we also need to approximate (Wesolowski and Weber 1996; Wesolowski et al. 1996) the non-additive part of the kinetic energy, $T_{s}^{\text{nad}}[\rho _{{A}} , \rho _{{B}}]$. For weakly interacting or non covalent interactions, there are sufficiently good density functional approximations available for this quantity. Employing these, the set of spinorbitals $\{\phi _{A}^{i}\}$ and $\{\phi _{B}^{j}\}$ in Eq. 11 can then be obtained by solving the modified Kohn–Sham equations with constrained electron density (KCSED) for each subsystem *A* and *B*. In this approach, each subsystem is solved individually with the other subsystem being included as a frozen environment (hence the alternative name frozen density embedding or FDE) in the form of an embedding potential, i.e. $\{\phi _{A}^{i}\}$ and $\{\phi _{B}^{j}\}$ are obtained as solutions of,

15 $$\begin{aligned}&\left[ \frac{-\nabla ^{2} }{2} + v_{\text{KS}}[\rho _{{A/B}}](\mathbf {r}) + v_{\text{emb}}[\rho _{{A}},\rho _{{B}}](\mathbf {r}) \right] \phi ^{i}_{{A}/{B}} (\mathbf {r},s)\nonumber \\&\quad =\epsilon ^{i}_{{A}/{B}} \phi ^{i}_{{A}/{B}} (\mathbf {r},s) \end{aligned}$$

where $\mathbf {r}$ and *s* are the spatial and spin coordinates, respectively. For simplicity, we assume that both subsystems have an even number of electrons so that we can integrate out the spin coordinate. The one-electron Kohn–Sham potentials are then

16 $$\begin{aligned} v_{\text{KS}}^{{A}/{B}}(\mathbf {r}) = v_{\text{nuc}}^{{A}/{B}}(\mathbf {r}) + \int \frac{\rho _{{A}/{B}(\mathbf {r})}}{\vert \mathbf {r} - \mathbf {r'}\vert } {\text{d}}\mathbf {r'} + \frac{\delta E_{\text{xc}}[\rho ]}{\delta \rho (\mathbf {r})} \Bigg \vert _{\rho (\mathbf {r}) = \rho _{{A}/{B}}(\mathbf {r})} \end{aligned}$$

and

17 $$\begin{aligned} v_{\text{emb}}^{{A}/{B}}(\mathbf {r})= & {} v_{\text{nuc}}^{{B}/{A}}(\mathbf {r}) + \int \frac{\rho _{{B}/{A}(\mathbf {r'})}}{\vert \mathbf {r} - \mathbf {r'}\vert } {\text{d}}\mathbf {r'} + \frac{\delta E_{\text{xc}}^{\text{nad}}[\rho _{{A}}, \rho _{{B}}]}{\delta \rho _{{A}/{B}(\mathbf {r})}}\nonumber \\&+ \frac{\delta T^{\text{nad}}[\rho _{{A}}, \rho _{{B}}]}{\delta \rho _{{A}/{B}(\mathbf {r})}} \end{aligned}$$

in which a non-additive exchange-correlation energy is defined for convenience:

18 $$\begin{aligned} E_{\text{xc}}^{\text{nad}}[\rho _{{A}} , \rho _{{B}}] = E_{\text{xc}}[\rho _{{A}} + \rho _{{B}}] - E_{\text{xc}}[\rho _{{A}}] - E_{\text{xc}}[\rho _{{B}}]. \end{aligned}$$

The above procedure can be made fully self-consistent by including the mutual polarization between the subsystems by the effect of the embedding potential $v_{\text{emb}}({r})$ in so-called freeze-and-thaw cycles (Wesolowski and Weber 1996; Gritsenko 2013; Jacob and Neugebauer 2014). Compared to other embedding schemes one may in this way account for polarization while still allowing for the Pauli repulsion that is missing in classical embedding (Jacob et al. 2006).

In applying these Kohn–Sham wave functions, there are two sources of non-orthogonality (Pavanello et al. 2013), the first one stemming from the overlap between the full electron diabatic states as in Eq. 10, and the second one arising at the subsystem level from the fact that the two set of orbitals $\{\phi _{A}^{i}\}$ and $\{\phi _{B}^{j}\}$ of subsystem *A* and *B* are non-orthogonal with respect to each other.

### Calculation of $\mathbf {H}^{\text{dia}}_{\text{LE}}$ block

Having discussed the calculation of the ground state, $\Phi _{\text{GS}}$ , by subsystem DFT in the previous section, we now introduce the formulas used in obtaining the matrix elements of the blocks $\mathbf {H}^{\text{dia}}_{\text{LE}}$, $\mathbf {H}^{\text{dia}}_{\text{CT}}$ and $\mathbf {H}^{\text{dia}}_{{\text{LE}}{-}{\text{CT}}}$ that we need in this work. We will compute these blocks independently (each block is computed without the knowledge of the other one) in separate sets of calculations. We assume that all of the LE states $\Phi _{A^{*}B}$ and $\Phi _{AB^{*}}$ in Eqs. 6 and 7 (Fig. 2) can formally be constructed from single-particle excitations from either $\psi _{0}^{A}$ or $\psi _{0}^{B}$:

19 $$\begin{aligned} \Phi _{A^{*}B}= & {} \vert \psi ^{A}_{1}\psi ^{B}_{0}\rangle \approx \sum _{i,a \in A} c^\mu _{ai}a_{a}^{\dagger }a_{i}\vert \Phi _{\text{GS}}\rangle \end{aligned}$$

20 $$\begin{aligned} \Phi _{AB^{*}}= & {} \vert \psi ^{A}_{0}\psi ^{B}_{1}\rangle \approx \sum _{j,b \in B} c^\nu _{bj}a_{b}^{\dagger }a_{j}\vert \Phi _{\text{GS}}\rangle \end{aligned},$$

where $a^{\dagger }$ and *a* are creation and annihiliation operators and indices are chosen such that $(i , j) \in \Phi _{\text{GS}}$ and $(a, b) \notin \Phi _{\text{GS}}$. The coefficients $c^\mu _{ai}$ and $c^\nu _{bj}$ describe the coupling of these single orbital transitions for state $\mu$ localized on *A* and state $\nu$ localized on *B*. The diagonal matrix elements of the $\mathbf {H}^{\text{dia}}_{\text{LE}}$ block are taken from two separate FDEu (where u denotes uncoupled) TDDFT (Casida and Wesołowski 2004; Neugebauer 2007, 2008, 2010; König et al. 2013) calculations by selecting the states of interest. In this work, we are interested in the lowest two states $Q_{y}$ and $Q_{x}$ for each of the chromophores. These energies are also called site energies in the context of interacting chromophores and are obtained as solutions of

21 $$\begin{aligned} \begin{pmatrix} {\textbf {A}}^{\text{sub}} &{} {\textbf {B}}^{\text{sub}} \\ -{\textbf {B}}^{\text{sub}} &{} -{\textbf {A}}^{\text{sub}} \\ \end{pmatrix} \begin{pmatrix} {\textbf {X}}^{\text{sub}} \\ {\textbf {Y}}^{\text{sub}}\\ \end{pmatrix} = \mathbf {\omega } \begin{pmatrix} {\textbf {X}}^{\text{sub}} \\ {\textbf {Y}}^{\text{sub}}\\ \end{pmatrix}, \qquad \text{sub} \in \bigg \{A^{*}B,AB^{*}\bigg \} \end{aligned}$$

Here, $\mathbf {A}^{\text{sub}}$ and $\mathbf {B}^{\text{sub}}$ is the subsystem partitioned response matrices (Neugebauer 2007; König et al. 2013) assuming real spatial orbitals, $\{\omega \}$ the set of eigenvalues (site energies) and $\{ \mathbf {X}^{\text{sub}},\mathbf {Y}^{\text{sub}} \}$ the set of corresponding excitation and de-excitation vectors for each of the $\Phi _{A^{*}B}$ and $\Phi _{AB^{*}}$ states, respectively [see for example Eq. 23 in Ref. König et al. (2013)]. Note that by choosing the FDEu starting ansatz the $\mathbf {H}^{\text{dia}}_{\text{LE}}$ matrix becomes diagonal inside the $\Phi _{A^{*}B}$ and $\Phi _{AB^{*}}$ subblocks. The coupling between the LE states $\Phi ^\mu _{A^{*}B}$ and $\Phi ^\nu _{AB^{*}}$, is subsequently approximated by the coupling between the transition densities of excitations localized on fragments *A* and *B* and yields the remaining non-zero elements of the $\mathbf {H}^{\text{dia}}_{\text{LE}}$ block. In the Tamm–Dancoff approximation (denoted as FDEc-TDA) it is given by König et al. (2013),

22 $$\begin{aligned} \langle \Phi ^\mu _{A^{*}B}\vert {\hat{H}}^{\text{el}}\vert \Phi ^\nu _{AB^{*}}\rangle \approx \int \int \rho _{A,\mu }(\mathbf {r})f(\mathbf {r}, \mathbf {r'})\rho _{B,\nu }(\mathbf {r'})\text{d}\mathbf {r}\text{d}\mathbf {r'}. \end{aligned}$$

where $\rho _{A,\mu }(\mathbf {r})$ and $\rho _{B,\nu }(\mathbf {r'})$ are the transition densities of excitations $\mu$ and $\nu$ corresponding to $\Phi _{A^{*}B}$ and $\Phi _{AB^{*}}$, respectively, and the kernel $f(\mathbf {r}, \mathbf {r'})$ is given by

23 $$\begin{aligned} {f}(\mathbf {r}_{1},\mathbf {r}_{2}) = \frac{1}{\vert \mathbf {r}_{2} - \mathbf {r}_{1}\vert } + \frac{\delta ^{2} E_{\text{XC}}[\rho ]}{\delta \rho (\mathbf {r}_{1}) \delta \rho (\mathbf {r}_{2})} \Bigg \vert _{\rho = \rho _{\text{tot}}} + \frac{\delta ^{2} T_{s}[\rho ]}{\delta \rho (\mathbf {r}_{1}) \delta \rho (\mathbf {r}_{2})} \Bigg \vert _{\rho = \rho _{\text{tot}}} \end{aligned}$$

where $E_{\text{XC}}[\rho ]$ and $T_s[\rho ]$ denote the usual exchange-correlation and kinetic energy functional and $\rho _{\text{tot}}$ is the summed density of subsystems *A* and *B*. The first term of Eq. 23 represents the Förster type Coulomb coupling between two localized excitations whereas additional short-range interactions are taken into account via the approximate exchange-correlation and kinetic energy functionals in the second and third term. The remaining elements to be considered are the overlap matrix elements, these are taken as elements of the unit matrix so that diagonalization of the LE submatrix will reproduce the FDEc-TDA results. We would like to note here that the above formulation of subsystem TDDFT lacks an explicit description of charge transfer excitations (which we calculate in the next section using explicitly constructed charge localized states) that become significant when two chromophores are sufficiently close to each other (Sen et al. 2021). Recent works by Neugebauer and co-workers using projection based embedding techniques (as an alternative to the conventional non-additive functionals used here) in combination with a supramolecular basis set in the context of subsystem TDDFT have been shown to be able to describe such inter-subsystem charge transfer excitations and reproduce the supramolecular results exactly (Tölle et al. 2019a, 2019b, 2020; Scholz et al. 2020; Tölle and Neugebauer 2022). We have not considered this extension of FDE here.

### Calculation of $\mathbf {H}^{\text{dia}}_{\text{CT}}$ block

A detailed discussion of the computation of CT energies with subsystem DFT and other approaches can be found in the literature (Pavanello and Neugebauer 2011; Pavanello et al. 2013; Solovyeva et al. 2014; Ramos et al. 2015). Here we will give the essential points, largely following Marcus Theory. We start by considering the charge transfer process in terms of (quasi-)diabatic charge localized states and first consider the particular case of $\Phi _{\text{CT1}}$ defined in Eq. 8 and describing the process

24 $$\begin{aligned} A + B \rightarrow A^{+} + B^{-} \end{aligned},$$

where an electron is transferred from *A* to *B*. In order to calculate the excitation energy $E^{\text{CT1}}$ of state CT1 ($A^{+}+B^{-}$) with respect to the GS ($A+B$) one then solves a 2 × 2 generalized eigenvalue equation (Pavanello and Neugebauer 2011; Pavanello et al. 2013; Migliore 2009, 2011). This yields two adiabatic state energies with their difference given by,

25 $$\begin{aligned} \Delta E^{\text{CT1}} = \sqrt{\frac{(H^{\text{dia}}_{11} - H^{\text{dia}}_{00})^{2}}{(1-(S_{01})^{2})} + 4V_{01}^{2}} \end{aligned},$$

where

26 $$\begin{aligned} V_{01} = \frac{1}{(1-(S_{01})^{2})} \left( H^{\text{dia}}_{01} - S^{01}\frac{H^{\text{dia}}_{00} + H^{\text{dia}}_{11}}{2}, \right) \end{aligned}$$

and we abbreviated CT1 by 1 and the GS by 0. $H^{\text{dia}}_{00}$, $H^{\text{dia}}_{11}$, $H^{\text{dia}}_{ab}$ and $S_{01}$ represents the diabatic energies of the GS and CT1, the coupling between them and their overlap, respectively. When the energy difference between the diabatic states is much larger than the coupling ($H^{\text{dia}}_{00} - H^{\text{dia}}_{11} \gg V_{01}$), the diabatic and adiabatic energies coincide, i.e. Eq. 25 at small overlap and coupling becomes,

27 $$\begin{aligned} \Delta E^{\text{CT1}} \approx H^{\text{dia}}_{11} - H^{\text{dia}}_{00} \end{aligned}$$

Similar considerations apply for the reverse CT state $\Phi _{\text{CT2}}$ defined in Eq. (9).

In the subsystem DFT framework, these CT states are constructed from broken-symmetry KS determinants. Orbitals are optimized following Eq. 15 in a series of freeze-and-thaw cycles, for two neutral fragments *A* and *B* comprising the ground state ($\Phi _{\text{GS}}$) and two charge-separated states $A^{+}$ and $B^{-}$ (or $A^{-}$ and $B^{+}$) comprising the product state $\Phi _{\text{CT1}}$ (or $\Phi _{\text{CT2}}$). These in total six sets of subsystem KS orbitals are combined to generate the three diabatic states $\Phi _{\text{GS}}$, $\Phi _{\text{CT1}}$ and $\Phi _{\text{CT2}}$. In contrast to the procedure followed for the LE states, where it is easy to consider many excited states as they all are generated from the same GS orbital set, the procedure for CT states will produce only one CT1 state and only one CT2 state, each with it own set of optimized orbitals that include the effect of polarization via the freeze–thaw optimization step.

For example, $\Phi _{\text{CT1}}$ in Eq. 8 is constructed as,

28 $$\begin{aligned}&\Phi _{\text{CT1}} = \vert \psi ^{A}_{+}\psi ^{B}_{-}\rangle \nonumber \\&\qquad \approx \frac{1}{\sqrt{(N_{A}-1)+(N_{B}+1)}}\bigg \Vert \phi _{A+}^{1}...\phi _{A+}^{i}...\phi _{A+}^{N_{A}-1}\phi _{B-}^{1}...\phi _{B-}^{j}..\phi _{B-}^{N_{B}+1}\bigg \Vert \end{aligned}$$

where $\phi _{A+}^{i}$, $\phi _{B-}^{j}$ are the set of orbitals for the corresponding charge localized cation $A^{+}$ and anion $B^{-}$. These states are then used to calculate the matrix elements in Eqs. 25 and 26 as,

29 $$\begin{aligned} H^{\text{dia}}_{01/2}= & {} E[\rho ^{(01/2)}(\mathbf {r})]S_{01/2} \end{aligned}$$

30 $$\begin{aligned} H^{\text{dia}}_{ii}= & {} E[\rho ^{(ii)}(\mathbf {r})] , \, i \in (0,1,2) \end{aligned}$$

Here $\rho ^{(01/2)}(\mathbf {r})$ is the transition density between states 0 and 1 or 2 (i.e. between $\Phi _{\text{GS}}$ and $\Phi _{\text{CT1}}$ or $\Phi _{\text{CT2}}$) and $S_{01/2}$ the corresponding overlap. $\rho ^{(ii)}(\mathbf {r})$ is the density calculated using orbitals of the appropriate diabatic states 0, 1 or 2 ($\Phi _{\text{GS}}$ or $\Phi _{\text{CT1}}$/$\Phi _{\text{CT2}}$). We need to account for two kinds of non-orthogonality in these calculations. Since the subsystem orbitals are the results of an FDE calculation, their product wave function is not necessarily normalized so that $S_{ii} \ne 1$. Furthermore, the off-diagonal elements $S_{01}$ and $S_{02}$ are in general non-zero ($S_{12}$ is zero, as mentioned before). This means that rather than focusing on only the blue part of Fig. 3, as is our intention, we also need to check the magnitude of the GS–CT coupling, or equivalently, the difference between the diabatic and adiabatic CT energies. As we will discuss later, we are in the current work in the weak coupling regime, which means that the desired $\mathbf {H}^{\text{dia}}_{\text{CT}}$ elements can be taken as the approximate energy difference Eq. 27 and we need not consider the GS explicitly.

We would like to point out here that the CT energies so computed using the subsystem formalism have the correct long-range behaviour, which the approximate functionals employed in conventional supramolecular TDDFT often fail to describe properly. The energy of a charge separated donor and acceptor in the subsystem formalism can be written as,

31 $$\begin{aligned} E_{A+....B-} = E_{A+} + E_{B-} + E_{\text{int}} \end{aligned},$$

where the $E_{A+}$ , $E_{B-}$ are the diabatic energies of the two ionized states and $E_{\text{int}}$ is the interaction energy between the two charged species given by,

32 $$\begin{aligned} E_{\text{int}}= & {} J[\rho _{A+}, \rho _{B-}] + V_{\text{nuc}_{A+}}[\rho _{B-}] + V_{\text{nuc}_{B-}}[\rho _{A+}] + V_{\text{nuc}_{A+}-\text{nuc}_{B-}} \nonumber \\&+E_{\text{xc}}^{\text{nad}}[\rho _{A+}, \rho _{B-}] + T_{s}^{\text{nad}}[\rho _{A+}, \rho _{B-}] \end{aligned}$$

At very long range, where there is zero overlap between $A^{+}$ and $B^{-}$, the non-additive terms drop out, while the other terms yield the correct limit: $E_{\text{int}} \approx - \frac{1}{R}$, where *R* is the distance between *A* and *B* (Solovyeva et al. 2014).

For the short-range or strong-coupling limit, we could in principle use Eq. 26 and include the GS explicitly in the calculation, but such an approach would require careful testing of the validity of the employed density functional approximation in this limit.

### Calculation of $\mathbf {H}^{\text{dia}}_{{\text{LE}}{-}{\text{CT}}}$ block

Having discussed the diagonal blocks of $\mathbf {H}^{\text{dia}}$, we now turn our attention to the coupling of the CT and LE states given by the $\mathbf {H}^{\text{dia}}_{{\text{LE}}{-}{\text{CT}}}$ block. The most rigorous approach would be to consider the broken-symmetry KS determinants of the preceeding section, as well as the full expansion of the TDA-TDDFT wave function, thereby accounting for the fact that these are constructed from different subsystem orbital sets and are not necessarily orthogonal. Here, we will introduce a simple approach using second quantization for non-orthogonal orbitals in order to calculate the necessary couplings. The approach described here represents a generalized form of the Fragment Orbital DFT (FODFT) approach previously proposed by Senthilkumar et al. (2003, 2005) for calculating charge transfer integrals for non-orthogonal orbitals and used later by Hernández-Fernández et al. (2016) for calculating electronic couplings for hole transfer in stacked porphyrin dyads.

In this approach we approximate the full interaction Hamiltonian by the fixed Kohn–Sham (KS) Hamiltonian matrix representation obtained in a supermolecular calculation. This Hamiltonian is diagonal in its eigenbasis of supermolecular orbitals and can be expressed in second quantization as:

33 $$\begin{aligned} \hat{H}^{\text{el}} \approx \hat{H}^{\text{KS}} = \sum _{p} \varepsilon _p a^{\dagger }_{p} a_{p} \end{aligned},$$

where $\{\varepsilon _p\}$ are the supermolecular orbital energies.

We then define a normal ordered Hamiltonian with the supermolecular ground state determinant $\Phi _{\text{GS}}$ as the reference vaccum,

34 $$\begin{aligned} \hat{H}_{N}= & {} \hat{H}^{\text{KS}}-\langle \Phi _0\vert H^{\text{KS}}\vert \Phi _0\rangle \nonumber \\= & {} \sum _a \varepsilon _a a^\dagger _a a_a - \sum _i \varepsilon _i a_i a_i^\dagger \end{aligned}$$

35 $$\begin{aligned}= & {} \sum _a \varepsilon _a b^\dagger _a b_a - \sum _i \varepsilon _i b_i^{\dagger } b_i \end{aligned},$$

where we have redefined the usual creation/annihilation operators ($a^{\dagger }$/*a*) in Eq. 34 in terms of hole/particle operators ($b^{\dagger }$/*b*) in Eq. 35 where the indices *i*, *a* denote the supermolecular occupied and virtual orbitals. We then construct a set of unpolarized reference fragment orbitals obtained from two independent fragment calculations of *A* and *B* which constitutes the above defined reference (supermolecular) vacuum (valid for weakly interacting fragments). Assuming that the excited states can be effectively described by single-particle excitation in this basis and that these underlying set of reference orbitals do not change, the LE state $\Phi _{A^{*}B}$ and CT state $\Phi _{\text{CT1}}$ in the hole/particle formalism can be written as,

$$\begin{aligned} \Phi _{\kappa _A}^{\alpha _A} \approx b_{\alpha _A}^{\dagger }b_{\kappa _A}^{\dagger }\vert 0\rangle \end{aligned}$$

and,

$$\begin{aligned} \Phi _{\lambda _A}^{\beta _B} \approx b_{\beta _B}^{\dagger }b_{\lambda _A}^{\dagger }\vert 0\rangle \end{aligned}$$

respectively. The Greek indices $\kappa _A$, $\lambda _A$ denote occupied orbitals on fragment *A* and $\alpha _A$, $\beta _B$ denote virtual orbitals on fragment *A* and *B*, respectively. The coupling between these two states is then given as (see SI for the complete derivation),

36 $$\begin{aligned} V^{A^{*}B,\text{CT1}}= & {} \langle \Phi _{\kappa _{A}}^{\alpha _{A}}\vert \hat{H_{N}}\vert \Phi _{\lambda _{A}}^{\beta _{B}}\rangle \nonumber \\&= \sum _{a} \varepsilon _a \delta _{\kappa \lambda }S'_{a\alpha _A}S'_{a\beta _B} - \sum _{i} \varepsilon _i S_{\alpha _A,\beta _B}S'_{i,\kappa _A}S'_{i,\lambda _A} \end{aligned},$$

where the elements of $\mathbf {S}$ and $\mathbf {S'}$ are defined as,

37 $$\begin{aligned} S_{\rho _B,\pi _A}= & {} \int \phi _{\rho _B}^{*}(\mathbf {x})\phi _{\pi _A}(\mathbf {x}) \text{d}\mathbf {x} \end{aligned}$$

38 $$\begin{aligned} S'_{p,\pi _A}= & {} \int \psi _{p}^{*}(\mathbf {x}) \phi _{\pi _A}(\mathbf {x}) \text{d}\mathbf {x} \end{aligned}$$

where $\psi _{p}$, $\phi _{\pi _A}$ and $\phi _{\rho _B}$ denote general orbitals of the supermolecule (*A* + *B*), fragment *A* and fragment *B*, respectively. Since we consider CT1 to occur from only the highest occupied molecular orbital of a fragment *A* ($H_A$) to the lowest unoccupied orbital of fragment *B* ($L_B$), for the two LE states on fragment *A*, namely $Q_{y}^{A}$ ( $H_{A} \rightarrow L_{A}$) and $Q_{x}^{A}$ ($H'_{A} \rightarrow L_{A}$), the coupling in Eq. 36 can be written out as,

39 $$\begin{aligned} V^{Q_y^A,\text{CT1}}= & {} \sum _{a} \varepsilon _{a} S'_{a,L_A}S'_{a, L_B} - \sum _{i} \varepsilon _{i} S_{L_A,L_B}{S'}^2_{i,H_A} \end{aligned}$$

40 $$\begin{aligned} V^{Q_x^A,\text{CT1}}= & {} - \sum _{i} \varepsilon _{i} S_{L_A,L_B}S'_{i,H'_A}S'_{i,H_A} \end{aligned},$$

where $H'_{A}$ and $L_{A}$ denote the HOMO-1 and LUMO on fragment *A*, respectively.

Similarly, the coupling between the states $\Phi _{A^{*}B}$ and $\Phi _{\text{CT2}}$ is given as,

41 $$\begin{aligned} V^{A^{*}B,\text{CT2}}= & {} \langle \Phi _{\kappa _A}^{\alpha _A}\vert \hat{H_{N}^{\text{el}}} \vert \Phi _{\lambda _B}^{\beta _A}\rangle \nonumber \\&= - \sum _{i} \varepsilon _i \delta _{\alpha \beta }S'_{i\kappa _A}S'_{i\lambda _B}+\sum _{a} \varepsilon _a S_{\kappa _A,\lambda _B}S'_{a,\alpha _A}S'_{a,\beta _A}. \end{aligned}$$

Given that CT2 occurs only from the highest occupied molecular orbital of a fragment *B* ($H_B$) to the lowest unoccupied orbital of another fragment *A* ($L_A$), the coupling with the two LE states on fragment *A* becomes,

42 $$\begin{aligned} V^{Q_y^A,\text{CT2}}= & {} -\sum _{i} \varepsilon _i S'_{i,H_A}S'_{i,H_B}+\sum _{a} \varepsilon _a S_{H_A,H_B}{S'}^2_{a,L_A} \end{aligned}$$

43 $$\begin{aligned} V^{Q_x^A,\text{CT2}}= & {} - \sum _{i} \varepsilon _i S'_{i,H'_A}S'_{i,H_B}+\sum _{a} \varepsilon _a S_{H'_A,H_B}{S'}^2_{a,L_A} \end{aligned}$$

Equations 39, 40 and 42, 43 along with the corresponding coupling terms for the LE states on fragment *B* (see SI, not shown here) form the eight unique couplings in which we recognize the contributions of hole as well as electron transfer, which are, respectively, governed by the overlap between the occupied and the virtual orbitals of the individual fragments.

Note that, in the above calculation of the couplings, we assumed the LE states ($Q_{y}$ and $Q_{x}$) to be pure states (i.e. composed of a single single orbital transition), thereby making their calculation fairly straightforward, obtained readily from a ground state Kohn–Sham calculation of $A+B$. In practice however, the LE states are composed of multiple single orbital transitions (see Eq. 19), in which case a more complete description of the couplings can be obtained either by a subsystem approach (Difley and Van Voorhis 2011) or by diabatization techniques (Voityuk and Rösch 2002; Yang and Hsu 2013; Hsu et al. 2008; Nottoli et al. 2018; Tölle et al. 2020). For the present purpose of the work, the above approximation is considered to be sufficient for a qualitative assessment.

A summary of the above approximations introduced in the context of the different methods and the structure of the matrix $\mathbf {H}^{\text{dia}}$ and $\mathbf {S}^{\text{dia}}$ used in Eq. 10 are shown in Table 1 and Fig. 3, respectively.

### Effect of a polarizable environment on $\mathbf {H}^{\text{dia}}_{\text{LE}}$, $\mathbf {H}^{\text{dia}}_{\text{CT}}$ and $\mathbf {H}^{\text{dia}}_{{\text{LE}}{-}{\text{CT}}}$ blocks

So far, we only consider the chromophores themselves, and not the effect of the surrounding protein matrix. This can be accounted for by means of an additional embedding potential, for which we selected the Discrete Reaction Field (DRF) model. The effect of a polarizable environment is in the DRF model accounted for in a molecular mechanics (MM) way via atomic point charges and static polarizabilities with a potential, $v^{\text{DRF}}$, given by Jensen et al. (2003a)

44 $$\begin{aligned} v^{\text{DRF}}({r_{i}}) = v^{\text{el}}({r_{i}}) + v^{\text{pol}}({r_{i}}) \end{aligned},$$

where the first term $v^{\text{el}}$ is the electrostatic operator and describes the Coulomb interaction between the QM system and the permanent charge distribution of the MM environment. The second term, $v^{\text{pol}}$, describes the induced polarization at the MM atoms due to interaction with the QM part and other MM atoms. These two terms are given by

45 $$\begin{aligned}&v^{\text{el}}({r_{i}}) = \sum _{s} \frac{q_{s}}{R_{si}} = \sum _{s} q_{s}T_{si}^{(0)},\\& v^{\text{pol}}({r_{i}})\nonumber = \sum _{s} \mu _{s,\alpha }^{\text{ind}} \frac{R_{si,\alpha }}{R_{si}^{3}} = \sum _{s} \mu _{s,\alpha }^{\text{ind}}T_{si,\alpha }^{(1)} \end{aligned}$$

with

46 $$\begin{aligned} T_{pq,\alpha _{1},...,\alpha _{n}}^{(n)} = \nabla _{pq,\alpha _{1}....}\nabla _{pq,\alpha _{n}}\left( \frac{1}{R_{pq}} \right) , \end{aligned},$$

where the interaction tensor to a given order, *n*, is given by $T_{pq,\alpha _{1}...,\alpha _{n}}^{(n)}$. $R_{pq}$ is the distance between the interacting entities, $R_{si,\alpha }$ is a component of the distance vector and $\mu _{s}^{\text{ind}}$ is the induced dipole at site *s*. The induced dipoles depend on the QM density and are updated during the self-consistent field cycles used to solve the QM part.

The DRF model has also been extended to the calculation of excited states in the linear response formalism of TDDFT where, an additional explicit response of the MM region DRF potential $v^{\text{DRF}}$ is accounted for in the kernel resulting from the response in the induced dipole upon change in the QM charge distribution. Similar to the calculation of the induced dipoles using the ground state density, in the excited state formalism it utilises the transition density for the calculation of the induced dipoles. It is defined by Jensen et al. (2003b) as

47 $$\begin{aligned} v_{\text{DRF}}[\delta \rho ](r_{i}, \omega ) = - \sum _{s} \mu _{s,\alpha }^{\text{ind}} [\delta \rho ](\omega )T_{si,\alpha }^{(1)} \end{aligned},$$

where $\mu _{s,\alpha }^{\text{ind}} [\delta \rho ]$ is the induced dipole at site *s* due to the perturbation in the density $\rho$ and $T_{si,\alpha }^{(1)}$ is the corresponding interaction tensor. In all our subsequent calculations we restrict ourselves to the adiabatic local density approximation (ALDA) of the kernel, so the dependence on $\omega$ is dropped from the above equations. In the presence of a DRF environment, the total effective Hamiltonian $\mathbf {H}^{\text{eff}}$ can be written as,

48 $$\begin{aligned} \mathbf {H}^{\text{eff}} = \mathbf {H}^{\text{el}} + \mathbf {H}^{\text{DRF}} \end{aligned},$$

where $\mathbf {H}^{\text{el}}$ is our block diagonal electronic Hamiltonian in the absence of the environment defined above, and $\mathbf {H}^{\text{DRF}}$ is the Hamiltonian describing the perturbation on each of the blocks of $\mathbf {H}^{\text{el}}$ due to the DRF environment. In the following section, we note the nature of perturbation of $\mathbf {H}^{\text{DRF}}$ on the blocks of the $\mathbf {H}^{\text{dia}}$ matrix

*Effect on* $\mathbf {H}^{\text{dia}}_{\text{LE}}$ *block* In the calculation of the matrix elements of the $\mathbf {H}^{\text{dia}}_{\text{LE}}$ block , the effect of the environment is accounted for in two steps—(1) As an additional potential term, $v^{\text{DRF}}$, given by Eq. 44 in the calculation of the ground state subsystem orbitals via Eq. 15 and (2) In the calculation of the diagonal (LE energies) and off-diagonal (LE couplings) matrix elements in Eq. 22 via the linear response of $v^{\text{DRF}}$ in Eq. 47.

*Effect on* $\mathbf {H}^{\text{dia}}_{\text{CT}}$ *block* The effect of the environment is explicitly accounted for in the calculation of the set of subsystem orbitals for the individual fragments, i.e. *A*($\{\phi _{A}^{i}\}$), *B*($\{\phi _{B}^{i}\}$), and $A^{+}$($\{\phi _{A+}^{i}\}$), $B^{-}$($\{\phi _{B-}^{i}\}$) or $A^{-}$($\{\phi _{A-}^{i}\}$), $B^{+}$($\{\phi _{B+}^{i}\}$) in the relevant diabatic states belonging to either the reactant or the product state via $v^{\text{DRF}}$ given by Eq. 44 and entering the ground state Kohn–Sham calculation in Eq. 15. It therefore affects the CT energy (i.e. the diagonal elements of this block) and the electronic coupling with the ground state through Eqs. 25 and 26.

*Effect on* $\mathbf {H}^{\text{dia}}_{{\text{LE}}{-}{\text{CT}}}$ *block* The effect of the environment is taken into account in the ground state Kohn–Sham Hamiltonian , ${\hat{H}}^{\text{KS}}$, in Eq. 33 of the combined system of $A + B$ as well in the calculation of the reference fragment molecular orbitals of *A* and *B* via $v^{\text{DRF}}$, given in Eq. 44.

## Computational details

**Fig. 4:**
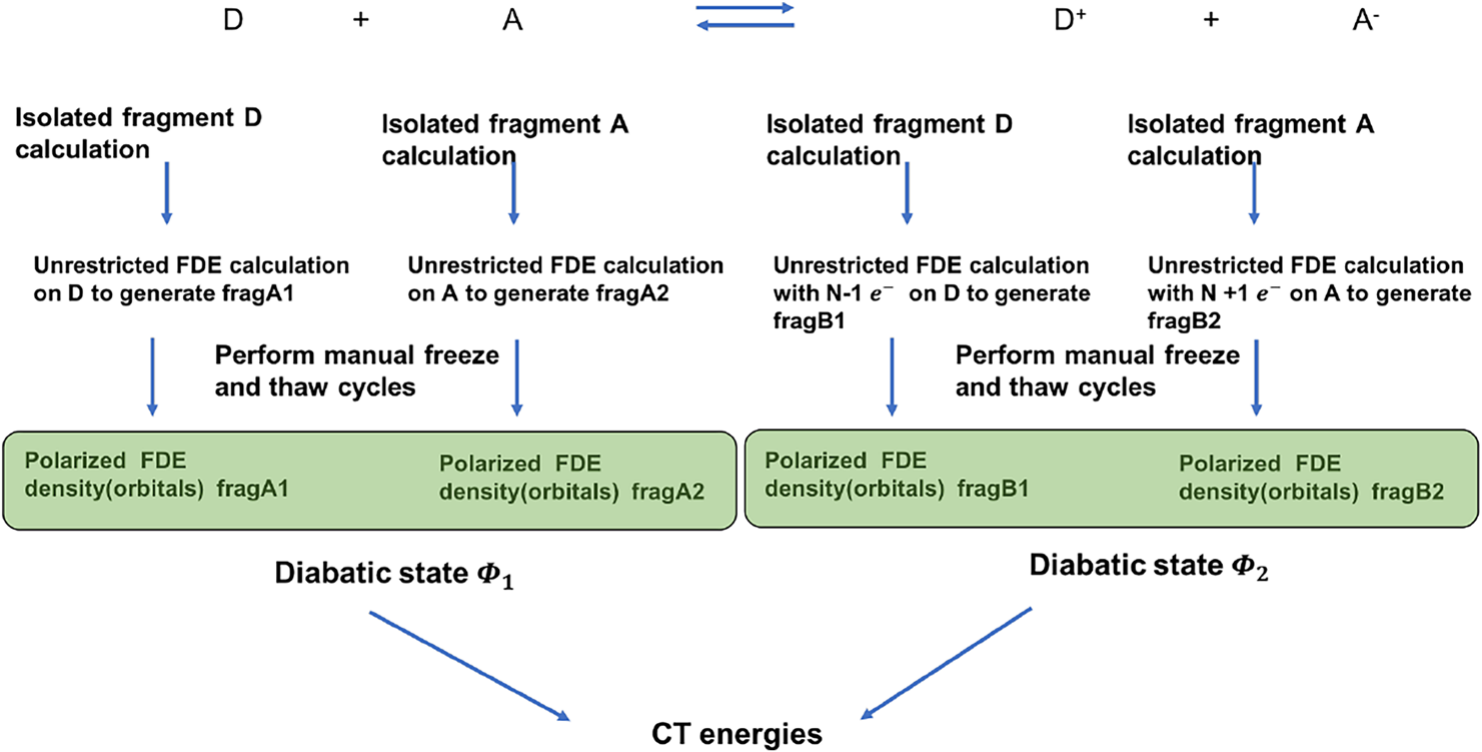
Workflow for the calculation of charge transfer (CT) state energies using frozen density embedding (FDE)

All calculations have been carried out with the Amsterdam Density Functional (ADF) program (Baerends et al. 2018; Te Velde et al. 2001) using the double-zeta polarised (DZP) basis set and the range-separated CAMY-B3LYP exchange and correlation (XC) functional. The CAMY-B3LYP XC functional is implemented with the XCFun library (Ekström et al. 2010, https://dftlibs.org/xcfun/) and is a modified version of the original CAM-B3LYP (Yanai et al. 2004) with a different switching function. The performance of this functional in conjunction with the basis set for the calculation of the *Q* band energies has shown reasonable agreements before (López-Tarifa et al. 2017) with Milne et al. (2015) and hence was used consistently throughout this work. For the Frozen Density Embedding (FDE) calculations, the GGA functional BLYP (Becke 1988; Lee et al. 1988) and PW91k (Lembarki and Chermette 1994) were used for the non-additive exchange-correlation and kinetic energy part of the embedding potential through out this work. For the calculation of the elements of $\mathbf {H}^{\text{dia}}_{\text{LE}}$ block, the Time-Dependent DFT (TDDFT) extension of the Frozen Density Embedding (FDE) scheme in the linear response regime (Neugebauer 2007; König et al. 2013) as implemented in ADF was used to calculate two lowest excitation ($Q_{y}$ and $Q_{x}$) for each of the chromophores, *Chla*611 and *Chla*612. In order to include the polarization effects, 3 freeze-and-thaw cycles were performed for each of the chromophores. The coupling between the local excitations was then subsequently calculated using the Tamm–Dancoff approximation in the FDEc formalism (König et al. 2013) as mentioned before. For the calculation of the diagonal elements of the $\mathbf {H}^{\text{dia}}_{\text{CT}}$ block, i.e. the CT energies, separate unrestricted calculations in the FDE framework were performed for each of the chromophores in the neutral ground state and the charge-separated state to generate a total of 4 unrestricted fragments. Three freeze-and-thaw cycles were performed for each of the fragment in order to introduce polarization effects. For the inter-subsystem contributions,we used the BLYP (Becke 1988; Lee et al. 1988) XC functional to evaluate Eqs. 29 and 30. A schematic overview of the workflow is shown in Fig. 4. The elements of the $\mathbf {H}^{\text{dia}}_{{\text{LE}}{-}{\text{CT}}}$ block were calculated using a recently developed stand-alone code Reduction of Orbital Space Extent (ROSE), specifically designed for localization of molecular orbitals. Senjean et al. (2021) The environment was modelled in the DRF framework as mentioned earlier, with Mulliken charges obtained from a Self-Consistent Charge Density Functional Tight Binding approach (Elstner et al. 1998) (SCC-DFTB) with third order corrections, using the parameter set 3ob-3-1 (Lu et al. 2015) as implemented within the AMS (Rüger et al. 2018) engine of ADF. The atomic polarizabilities were taken from the standard Thole’s set of atomic polarizabilities (Thole 1981; Van Duijnen and Swart 1998) as given inside ADF. [In addition, for the Magnesium and Phosporous atom, polarizability values were taken from Ref. Stout and Dykstra (1995) and Lupinetti and Thakkar (2005), respectively]. All the elements of the $\mathbf {H}^{\text{dia}}$ matrix were calculated on a total of 103 frames with 51 in the first set and 52 in the last set separated by 400 ps in each window, from the classical trajectory [labelled as trajectory “A” in the original nomenclature by Ligouri et al in reference (Liguori et al. 2015)] computed using GROMACS Molecular Dynamics package (Van Der Spoel et al. 2005). Such a separation of the frames was motivated by the conformational changes observed in the particular selected trajectory between *Chla*612 and *Chla*611 towards the end of the simulation in reference (Liguori et al. 2015). The input geometric structure from each frame of the trajectory for the QM calculations was prepared in two steps. First, we use the visualization program VMD (Humphrey et al. 1996) to visualize and select chromophores *Chla*612 and *Chla*611 and the neighbouring residues (protein, chromophores and lipids) within a distance of $\approx$ 7 Å from each of the chromophore porphyrin rings in the first and last set of snapshots of the trajectory and save the coordinates for the resulting snapshots. In the second step, we process each of the frames using the RDKit (http://www.rdkit.org) library, wherein the DRF environment is separated from the QM region consisting of *Chla*612 and *Chla*611, followed by the addition of all the missing hydrogens, correction of the bond order of the porphyrin ring of the two chlorophylls and removal of the phytol chains of the chlorophylls in order to reduce the computational cost [removal of the phytol chain have been shown previously to not significantly affect the site energy calculations (López-Tarifa et al. 2017)]. Thereafter, we calculate all the elements of the $\mathbf {H}^{\text{dia}}$ in three different sets of calculations for each frame, for the $\mathbf {H}^{\text{dia}}_{\text{LE}}$, $\mathbf {H}^{\text{dia}}_{\text{CT}}$ and $\mathbf {H}^{\text{dia}}_{{\text{LE}}{-}{\text{CT}}}$ blocks, respectively, as mentioned above. The generation of the input geometries and the corresponding preparation of inputs for the subsequent ADF calculation were done in a fully automatised way using the Python Library PLAMS (Handzlik et al. 2018) as interfaced with ADF. As a final note, we would to mention that the numerical costs are dominated by the (not well optimized) calculation of the $\mathbf {H}^{\text{dia}}_{\text{CT}}$ block which takes 17 h of the 20 h in total needed per snapshot on one node (with 128 cores) of the Dutch supercomputer Snellius.

## Results and discussion

### The supramolecular picture

Förster resonance energy transfer (FRET) (Förster 1948) and Marcus electron transfer (ET) (Marcus and Sutin 1985) processes are both usually described in terms of local and non-local diabatic states. This is straightforward in subsystem DFT, but also in supermolecular TDDFT with approximate exchange-correlation functionals, the resulting adiabatic states can sometimes still be interpreted in a diabatic picture. This is especially so if the local Frenkel (LE) type excitons and the charge-separated (CT) states are energetically well separated. This identification is difficult, however, when low-lying CT states, occurring either as an artefact of the method itself or otherwise, start to mix with these local excitations. In a previous work (Sen et al. 2021) on the *Chla611–Chla612* dimer, we studied such a mixing of the CT states upon decreasing the distance between the two chlorophylls from 9 to 5 Å. In that work we used a supermolecular approach but quantified the amount of mixing in terms of a charge transfer number (Plasser and Lischka 2012) which indicated a sharp increase of LE–CT mixing upon decreasing the interchromophoric distance. However, due to the delocalized nature of the Highest Occupied and Lowest Unoccupied Molecular Orbitals (HOMO and LUMO) for that conformer, a clear interpretation in terms of diabatic LE and CT states remained elusive. In this work we provide such an interpretation and construct the individual matrix elements of $\mathbf {H}^{\text{dia}}$ to get a better picture of the couplings and energetics of these diabatic states. In the next section, we focus on the individual matrix elements of $\mathbf {H}^{\text{dia}}$ for selected snapshots of the MD trajectory spanning the beginning (sampled by first set of snapshots) of the simulation where the *Chla611* and *Chla612* chromophores are still far apart as well as the end of the simulation (sampled by last set of snapshots) where they are relatively close.

### The diabatic picture

#### Local excitation (LE) and charge transfer (CT) energies

**Fig. 5:**
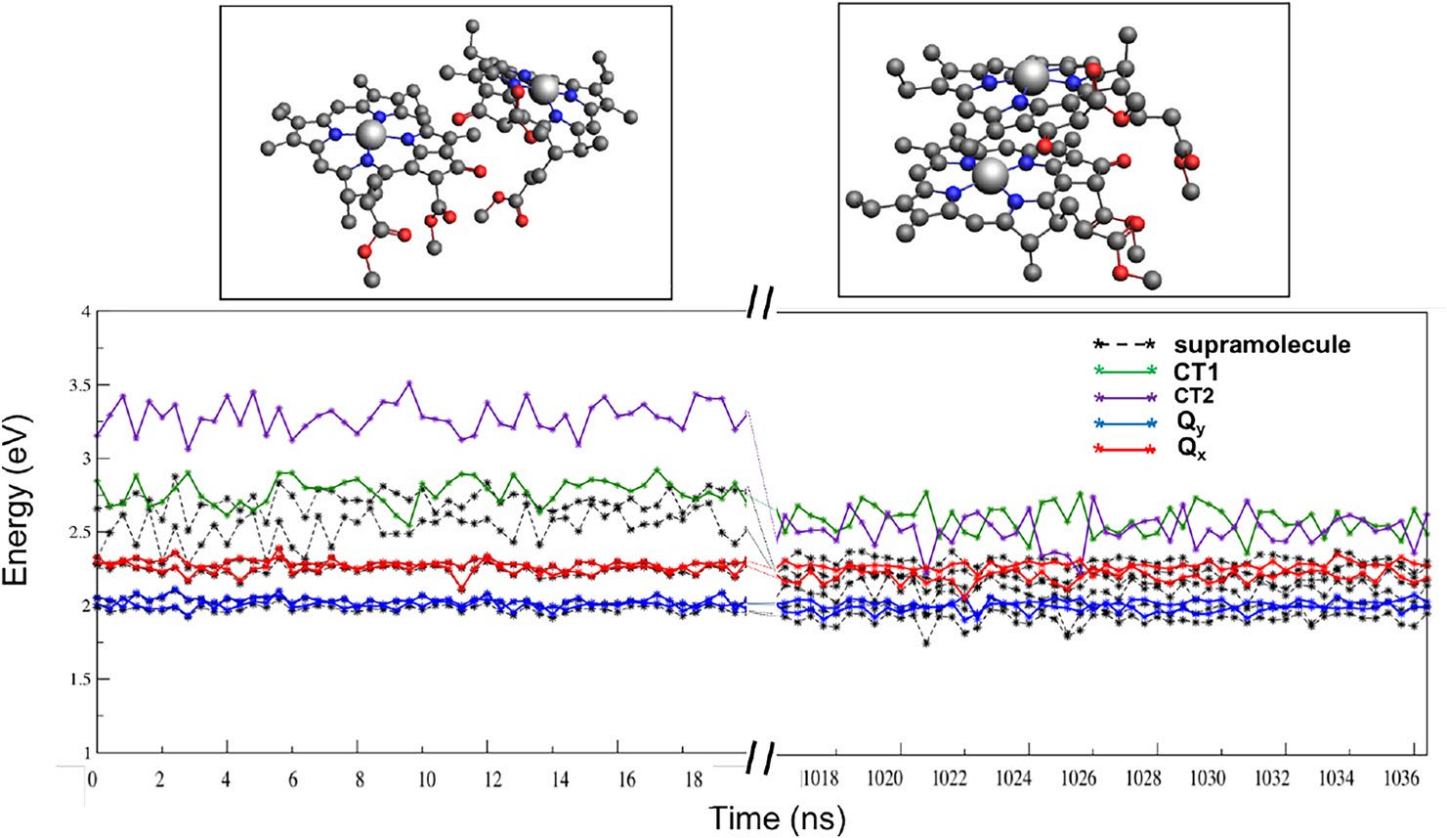
The 4 LE and 2 CT diabatic states of the *Chla612–Chla611* dimer are shown for the initial and final (left and right of the split on *x*-axis) parts of the trajectory. For comparison we also show with black dotted lines the lowest six states obtained in a supramolecular calculations . All units are in eV. Also shown in boxes are the two structures of the *Chla612–Chla611* dimer from the beginning (left) and end (right) of the trajectory

In Fig. 5 we show the four relevant ($Q_{x1}$, $Q_{y1}$, $Q_{x2}$, and $Q_{y2}$) local excitation energies of the two chlorophylls (*Chla612* and *Chla611*) along with two lowest CT state energies, CT1 ($Chla611^{+}/Chla612^{-}$) and CT2 ($Chla612^{+}/Chla611^{-}$), which represent the diagonal elements of the blocks $\mathbf {H}^{\text{dia}}_{\text{LE}}$ and $\mathbf {H}^{\text{dia}}_{\text{CT}}$ of the diabatic matrix respresentation (Fig. 3).

To quantify these values we also list the averaged LE and CT energies in Table 2. As can be seen from Fig. 5 and Table 2, the LE energies are minimally effected (the lowest $Q_{y2}$ is red-shifted by $\approx$ 0.02 eV) by the changed orientation and reduction of dimer distance in the chromophore pair. These energies are also relatively robust with respect to the smaller geometry fluctuations occuring between different snapshots with a variance of only 0.001 eV. The CT (CT1 and CT2) energies on the other hand show both a much larger fluctuation between snapshots and a clear decrease in energy going from the more distant geometries in the first set of snapshots to the close arrangement in the last set. The fluctuations of the CT energies are not unexpected as CT states have substantially larger reorganization energies than the local excitations as the Franck–Condon point is further away from the minimum than is the case for LE states. Looking closer at the overall trend for these CT energies we note the drop in energies of both of the CT states that we anticipated from the earlier supermolecular analysis. We see in Fig. 5 both the CT energies coming down and even crossing the $Q_{x}$ line at some points. In addition we observe that the relative gap between the two CT states decreases from an average of $\vert \Delta E_{\text{CT}}\vert$ of 0.51 to only 0.07 eV. The latter observation can be explained by the asymmetric orientation and thereby polarization (as represented by the DRF embedding potentials) for CT1 and CT2 in the beginning of the trajectory. In the final part of the studied trajectory the arrangement of the chromophores is more symmetrical and the polarization in the CT states is more similar, thereby bringing the energies of these two CT states closer together. Still, with the absence of an exact symmetry, the two CT states are not fully equivalent also in this stage.

**Table 2:** The LE and CT energies $\langle E\rangle$ and their variance $\sigma ^{2}$ averaged over the first and the last set of frames of the trajectory

First50			Last50		CRYS
	$\langle E \rangle$	$\sigma ^{2}$	$\langle E \rangle$	$\sigma ^{2}$	
$Q_{y1}$	2.02	0.001	2.03	0.001	2.07
$Q_{y2}$	2.01	0.001	1.99	0.001	2.02
$Q_{x1}$	2.27	0.001	2.28	0.001	2.36
$Q_{x2}$	2.26	0.002	2.21	0.002	2.34
*CT*1	2.77	0.008	2.59	0.009	2.83
*CT*2	3.28	0.011	2.52	0.012	3.28

The values for the geometry in the crystal are shown in the last column. All units are in eV

From Eq. 31, the CT1 and CT2 energies, $\vert \Delta E_{\text{CT}}\vert$ not only depend on the absolute energies of the charge localized fragments (i.e. $E_{A+} + E_{B-}$ and $E_{A-} + E_{B+}$) but also on their interaction energies $\Delta E_{\text{int}}$. The latter, negative, contribution to the CT energy is the driving force for the lowering of the CT energy towards the end of the trajectory. This enhances the mixing of the CT states with the local states and makes switching to an adiabatic picture by either diagonalizing the diabatic matrix representation or by using supermolecular TDDFT necessary. In the first part of the trajectory, the adiabatic (black) curves align well with the diabatic LE states, with only for the CT states that are hard to describe by density functional approximations significant deviations visible. For the structures from the final part of the trajectory, also the lowest supermolecular state is clearly below the lowest diabatic LE state, corroborating the influence of CT states in lowering this energy.

**Fig. 6:**
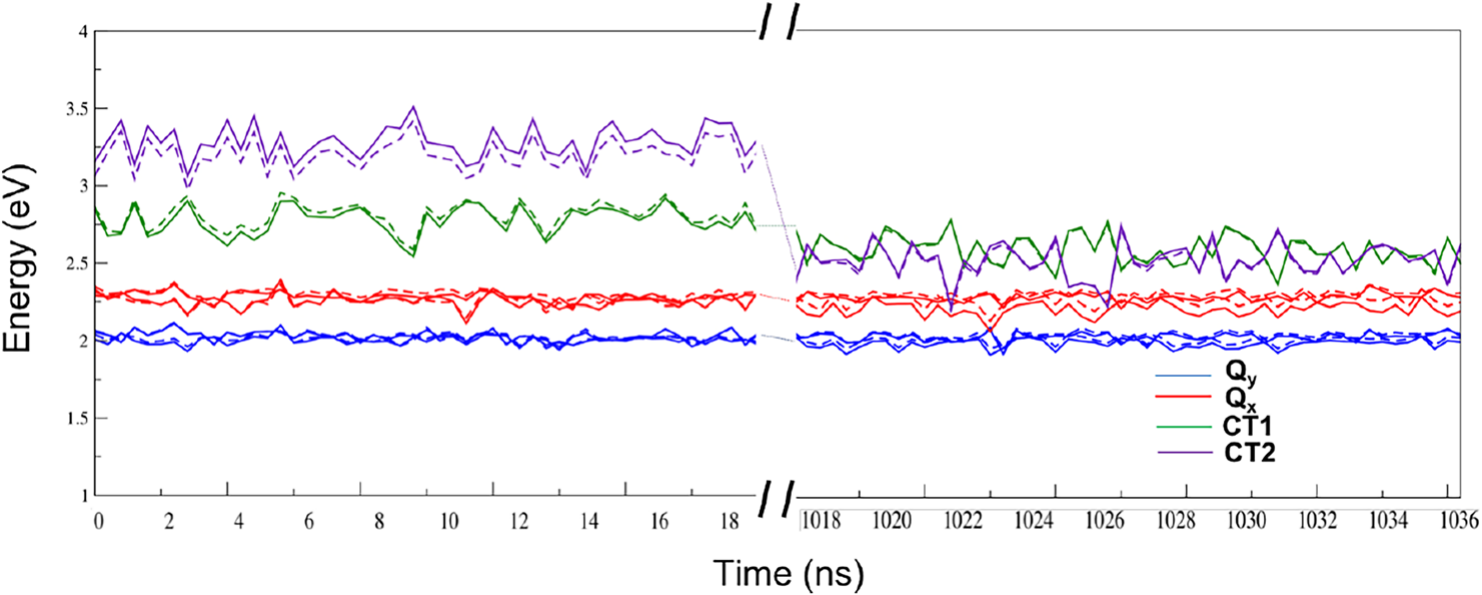
The effect of the DRF environment on the 4 LE states and 2 CT states in the snapshots taken from the first and last sets of the trajectory (left and right of the split on the *x*-axis). Bold lines denote DRF environment and dotted lines denote vacuum calculation for the *Chla612–Chla611* dimer. All units are in eV

*Effect of the environment on LE and CT energies* In order to assess the effect of the protein environment on the LE and CT energies we repeated the calculation of LE and CT energies of the dimer in vacuum. Figure 6 shows the effect of the environment on the diabatic states in the beginning and at the end of the trajectory. The average site energies and the CT energies with and without the environment are shown in Table S1 in the supplementary information. We note that: (i) all the averaged site energies are red-shifted by the environment, both in the beginning and at the end of the trajectory, with the exception of the $Q_{y2}$ and $Q_{x2}$which remain unaltered in the beginning of the trajectory. (ii) The CT energies are most strongly influenced in the beginning of the trajectory, with both the CT energies shifted by the environment in opposite directions (CT1 red-shifted and CT2 blue-shifted) thereby increasing the average gap between them. This effect can be explained by the asymmetric distribution of partial charges in the immediate proximity of both the chlorophylls (in particular the DPPG ligand near *Chla*611) in the beginning of the trajectory that we already mentioned in the preceding section. Towards the end of the trajectory, on the other hand, the stacked and symmetric arrangement of the chlorophylls with a reduced interchromophore distance, renders the two charge-separated states less sensitive to external perturbations. Looking at the magnitude of the environment shifts we conclude that overall the environment plays a minor role in determining the energies of the LE and CT states.

#### Coupling between the diabatic states

**Fig. 7:**
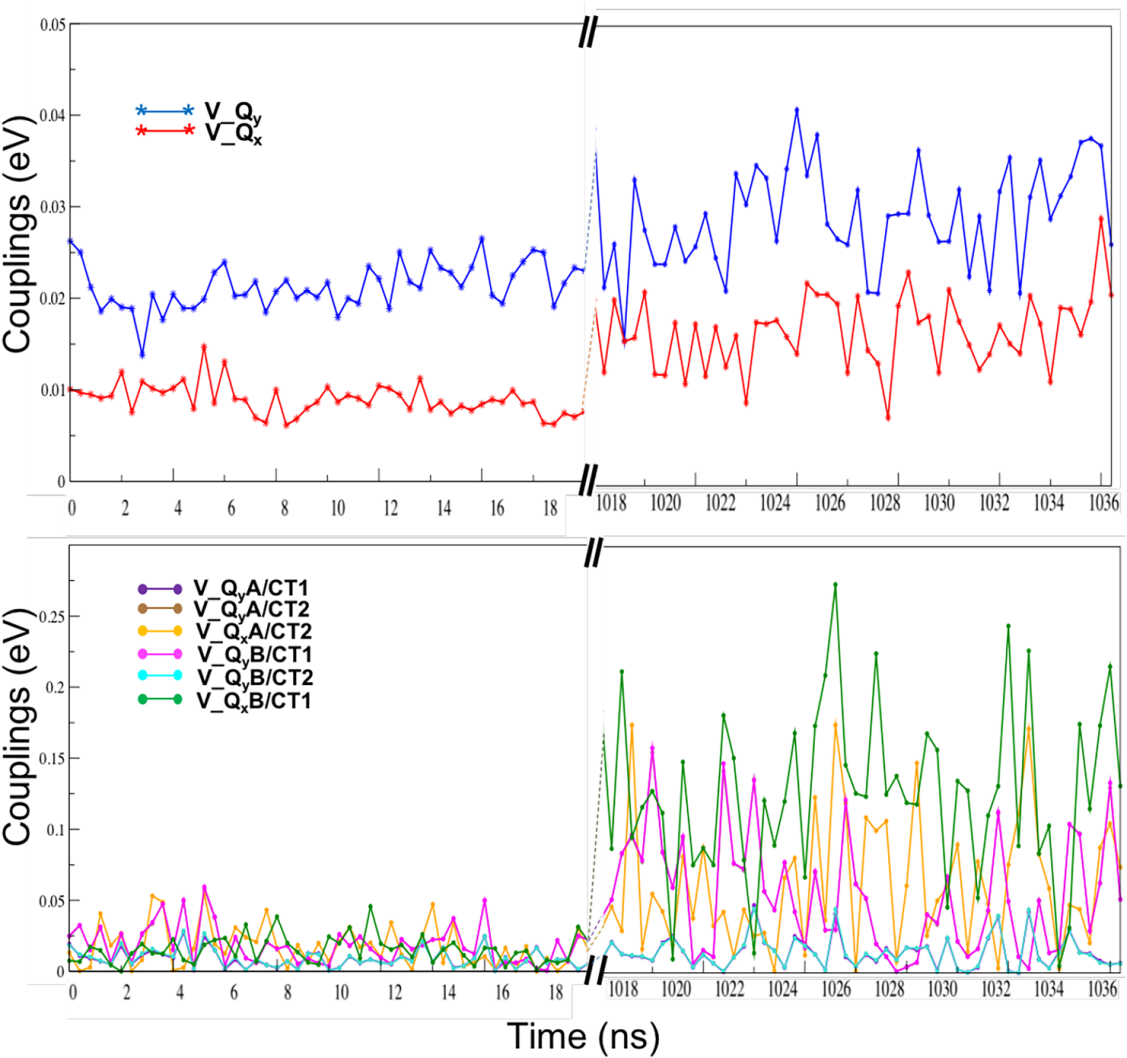
The absolute LE/LE couplings between $Q_{y}$ and $Q_{x}$ ($V_{Qy}$ and $V_{Qx}$) (upper panel) and the LE/CT couplings corresponding to hole and electron transfer (lower panel, see text for notations) from the first and last set of frames (left and right of the split on *x*-axis) of the trajectory are shown. All units are in eV

We now consider the couplings between the diabatic states (off-diagonal elements of $\mathbf {H}^{\text{dia}}$, see Fig. 3) that determine the final adiabatic picture. The off-diagonal elements in the LE block represent the predominantly Coulomb-like coupling between the local excitations which are responsible for Förster resonant energy transfer (Förster 1948), whereas the elements in the LE/CT block represent the overlap-dependent couplings between the local and CT states, which play an equally important role in Marcus electron transfer theory (Marcus and Sutin 1985). We note that these LE/CT couplings can describe both electron transfer between the fragment LUMOs (one coupling element), as well as hole transfer involving the two sets of HOMO and HOMO-1 located on each fragment (three possibilities), excluding transfer between the two HOMO-1 orbitals that is of minor importance). Table 3 and Figure 7 show the values of all the relevant LE/LE and LE/CT coupling elements: between the two LE $Q_{y}$ states ($V_{Q_{y}}$), the two LE $Q_{x}$ states ($V_{Q_{x}}$), and between these and the two possible CT states CT1 and CT2, as discussed in the previous sections: $V^{Q_{y}^{A},\text{CT1}}$,$V^{Q_{y}^{A},\text{CT2}}$, $V^{Q_{x}^{A},\text{CT2}}$, (and $V^{Q_{y}^{B},\text{CT1}}$, $V^{Q_{y}^{B},\text{CT2}}$, $V^{Q_{x}^{B},\text{CT1}}$), where *A* and *B* are *Chla611* and *Chla612*, respectively. Note that the coupling $V^{Q_{x}^{A},\text{CT1}}$ (and $V^{Q_{x}^{B},\text{CT2}}$) is negligible owing to the mutual orthogonality of the HOMO and HOMO-1 for each of the fragments *A* and *B* (see Eq. 40) and is not shown.

**Table 3:** The average of the absolute LE/LE and LE/CT couplings $\langle E\rangle$  and their variance $\sigma ^{2}$ averaged over the first and the last set of frames of the trajectory

First50			Last50		CRYS
	$\langle |V| \rangle$	$\sigma ^{2}$	$\langle |V| \rangle$	$\sigma ^{2}$	
$V_{Qy}$	0.021	6.2 × $10^{-6}$	0.029	3.1 × $10^{-5}$	0.026
$V_{Qx}$	0.009	2.8 × $10^{-6}$	0.017	1.6 × $10^{-5}$	0.005
$V_{Q_{y}A/\text{CT1}}$	0.009	4.3 × $10^{-5}$	0.014	1.2 × $10^{-4}$	0.005
$V_{Q_{y}A/\text{CT2}}$	0.019	1.8 × $10^{-4}$	0.054	1.6 × $10^{-3}$	0.019
$V_{Q_{x}A/\text{CT2}}$	0.017	2.2 × $10^{-4}$	0.062	2.0 × $10^{-3}$	0.015
$V_{Q_{y}B/\text{CT1}}$	0.019	1.9 × $10^{-4}$	0.055	1.6 × $10^{-3}$	0.020
$V_{Q_{y}B/\text{CT2}}$	0.009	4.5 × $10^{-5}$	0.014	1.2 × $10^{-4}$	0.007
$V_{Q_{x}B/\text{CT1}}$	0.015	9.1 × $10^{-5}$	0.128	3.4 × $10^{-3}$	0.014

The values for the geometry in the crystal are shown in the last column. All units are in eV. See text for notations

From Fig. 7 the much smaller variation for the LE/LE coupling compared to the LE/CT coupling stands out. The variation of the LE/LE coupling with the interchromophore distance *R* can be qualitatively described by an interacting dipole model (Liguori et al. 2015; López-Tarifa et al. 2017) and exhibits a $\sim R^{-3}$ (dipole–dipole) dependence. This can be contrasted to the exponential dependence $\sim e^{-\beta R/2}$ on this distance that is applicable for the LE/CT coupling (Cave and Newton 1996). Both couplings are enhanced in the final part of the studied trajectory in which interchromophoric distances are much smaller than that in the initial frames.

**Fig. 8:**
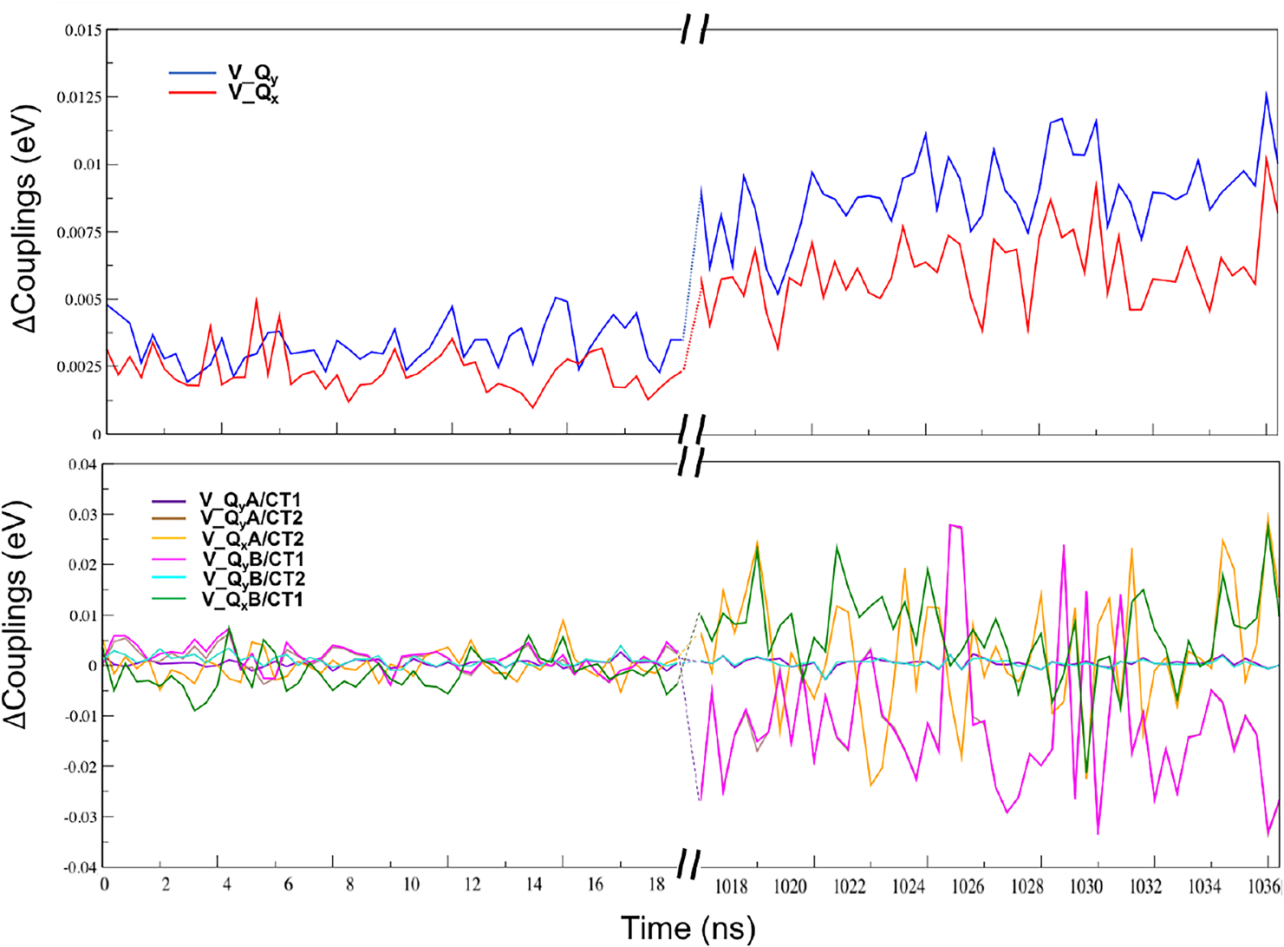
The difference of the LE/LE couplings involving $Q_{y}$ and $Q_{x}$ ($V_{Qy}$ and $V_{Qx}$, upper panel) and LE/CT couplings (lower panel, see text for notations) between DRF and vacuum ($V_{\text{DRF}} - V_{\text{VAC}}$), $\Delta \text {Couplings}$, for the first and last set of frames (left and right of the split on *x*-axis) of the trajectory are shown. All units are in eV

*Effect of environment on the couplings* The environmental effect on the LE/LE and the LE/CT couplings are shown in Fig. 8 as differences in the absolute couplings. Table S2 of the supplementary information also lists the averaged couplings in vacuum and in DRF environment. In case of non-orthogonal orbitals, the LE/CT couplings are not only determined by the different overlap terms, but also by the supramolecular KS orbital energies (see section on calculation of $\mathbf {H}^{\text{dia}}_{{\text{LE}}{-}{\text{CT}}}$ block). The variations of these LE/CT couplings upon inclusion of the environment are a direct consequence of the effect of the environment on both of these quantities. A further detailed analysis of this effect is beyond the scope of this current work. For the LE/LE couplings we see both for the $Q_{y1}$, $Q_{y2}$ and $Q_{x1}$, $Q_{x2}$ a more significant effect than seen previously for their energies. The increase in coupling upon including the environment effects (so relative to a vacuum treatment) can be explained by the increase of the magnitude of the transition dipole moments of all the local excitations. ($Q_{y1}$, $Q_{y2}$ and $Q_{x1}$, $Q_{x2}$; see Fig. S1 in supplementary information). This explicit treatment of the environment makes it possible to avoid the often applied scaling of transition dipole moment to achieve better agreement with experimental data. As such scaling does not provide additional insights we do not apply this in the present work. We note that our results here are in reasonable agreement with the recent works of Sláma et al. (2020) with minor differences arising from the different levels of theory and MD simulations used in both works. For comparison of the LE/LE couplings using the FDEc-TDA approach for the lowest $Q_{y}$ bands to other methods, we refer to López-Tarifa et al. (2017) as well as earlier work (Scholes et al. 1999; Frähmcke and Walla 2006; Madjet et al. 2006; Kenny and Kassal 2016; Müh et al. 2010; Chmeliov et al. 2015; Duffy et al. 2013) on treating these couplings in the most efficient way.

### The adiabatic picture

**Fig. 9:**
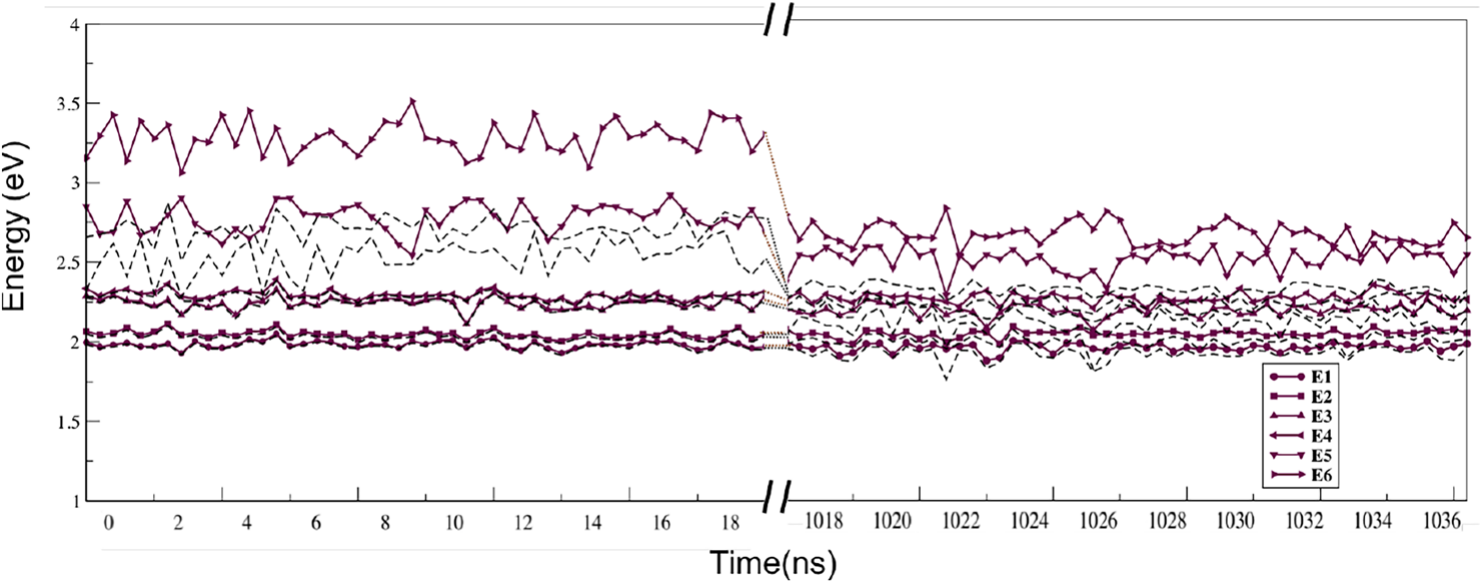
The six adiabatic states labelled E1–E6 are shown in bold in increasing order of their energy from the beginning and the end (left and right of the split on *x*-axis) of the trajectory. Also shown in dotted lines are the lowest six supramolecular states. All units are in eV

With the energy gap between the LE and the CT states decreased and their coupling increased at shorter distances (the last part of the trajectory), we expect to see an increased mixing between the CT states with the LE states. It had been shown previously from a disordered averaged exciton model, that such coupling of CT states to LE states can strongly modulate the optical spectra (Cupellini et al. 2018). Such a pronounced effect of the mixing of the CT states with the LE states at the end of the trajectory can indeed be seen when switching to the adiabatic picture. Solving for the generalized eigen value problem (Eq. 9) in the basis of the six diabatic states ($Q_{y1}, Q_{x1}, Q_{y2},Q_{x2}, \text{CT1}$ and CT2) for the first and last set frames of the trajectory, shows an average red-shift of about 0.02 eV of the lowest state. This state acquires clear mixed LE/CT character towards the end of the trajectory which is in qualitative agreement with the earlier supermolecular analysis. This indicates that the composite manner of constructing the matrix that is to diagonalized is adequate. To compare better with the supermolecular picture we show both the new set of adiabatic states as well as the six lowest supermolecular excitations in Fig. 9. Like already seen in the diabatic picture (Fig. 5), where there is little interaction between the CT states and the local states, the lowest lying adiabatic states follow closely to those of the supramolecular states and retain their local character. The two highest lying adiabatic and supramolecular states on the other hand, represent states which are predominantly CT in character, and get significantly different energies in the two approaches. The supermolecular ones are hereby likely to be to low in energy due to flaws in the treatment of CT states with the available density functional approximations where the adiabatized states of the current approach could be too high due to missing couplings with states at higher energies.

Nonetheless, this study provides a qualitative assessment of the importance of accounting for CT states and electron delocalization when studying the energy landscape of pigment-protein complexes, particularly in the case of strongly interacting pigments. Furthermore, it shows how pigment re-organizations following protein conformational changes can greatly affect the spectral properties. This spectral tunability is fundamental for LHCs to be able to switch between different functional states. Moreover, an improved knowledge of the nature of these dimer excitations in different orientations can provide hints to understand the effects of artificial modifications of the Chla611–Chla612 binding pocket on the overall absorption spectrum of the complex.

## Conclusion

In this work, we looked into changes in energies of the low-lying states of the chlorophyll dimer Chla611–Chla612 induced by a conformational change of the dimer in the highly disordered terminal emitter domain reported previously (Liguori et al. 2015). In order to get a better insight into these states, we elucidate the effect of this conformational change on the LE and CT state energies of the dimer along with their corresponding couplings using subsystem DFT calculations on snapshots from a $\mu$*s* MD simulation generated previously. Subsystem DFT provides an alternate localized diabatic approach as compared to the delocalized picture arising from supramolecular DFT, providing an unambiguous identification of the local and CT states. We find that the re-orientation of Chla611–Chla612 dimer upon equilibration brings down the energies of the Chl–Chl CT states, causing an increase in the mixing of the CT and LE states (which remain almost unaffected), accompanied by an increased coupling between them. This, in combination with an increase in the coupling between the local states themselves, facilitated by an reduced interchromophoric distance, gives rise to red-shifted low-lying mixed excitonic/CT states. Previous studies have shown that such mixing of low-lying charge transfer states with the $Q_{y}$ band can be operative in the the *Chla* band (Romero et al. 2009; Ramanan et al. 2017; Novoderezhkin et al. 2016; Chmeliov et al. 2016) and are indicative of the so-called ‘red-emitting states’ in the fluorescence spectra as observed in aggregates, supported by femtosecond Transient Absorption(TA) kinetics and Stark spectroscopy studies (Kell et al. 2014; Krüger et al. 2010; Müller et al. 2010). Moreover, Chl–Chl CT states have also been shown to play an important role in regulating energy flow through antenna complexes and create energy sinks in the reaction centers (RC) of photosynthetic complexes (Madjet et al. 2009; Raszewski et al. 2008; Novoderezhkin et al. 2005, 2007). Our study therefore provides further evidence that low-lying CT states, prompted by a favourable conformational change of the chlorophyll dimer, can play an pivotal role in regulating light harvesting and can create energy sinks facilitated by an increased excitonic interaction.

## Supplementary Information

Below is the link to the electronic supplementary material.

Supplementary file1 (PDF 453 kb)—Derivation of the LE–CT couplings; Table S1 and S2 for effect of environment on excitation energies and couplings; Figure S1 for effect of environment on transition dipoles.

## Data Availability

The data from this work are available upon reasonable request.
