# Supplementary material for: Towards the description of charge transfer states in solubilised LHCII using subsystem DFT
Source: Photosynth Res. 2022 Aug 21;156(1):39–57. doi: 10.1007/s11120-022-00950-7 (PMC10070235; doi:10.1007/s11120-022-00950-7)
Supplement: Supplementary file 1 — Supplementary file1 (PDF 453 kb)—Derivation of the LE–CT couplings; Table S1 and S2 for effect of environment on excitation energies and couplings; Figure S1 for effect of environment on transition dipoles. [file 11120_2022_950_MOESM1_ESM.pdf]

Supporting Information

Towards the description of Charge Transfer States in Solubilised LHCII using Subsystem DFT

Souloke Sen and Lucas Visscher*

Division of Theoretical Chemistry, Faculty of Sciences, Vrije Universiteit Amsterdam, De Boelelaan 1083,Amsterdam,1081 HV,The Netherlands

E-mail: l.visscher@vu.nl

Table of Contents:

1. Derivation of the LE/CT couplings Pg. S2
2. Effect of environment on the Energies and Couplings Pg. S5
3. Effect of environment on Transition Dipoles Pg. S6

Derivation of the LE/CT couplings

In this derivation we approximate the full interaction Hamiltonian by the fixed Kohn-Sham Hamiltonian matrix representation obtained in a supermolecular calculation. This Hamiltonian is diagonal in its eigenbasis of supermolecular orbitals $\{\psi\}$ and can be expressed in second quantization as:

$$\hat{H}^{el} \approx \hat{H}^{KS} = \sum_p \varepsilon_p a_p^\dagger a_p \tag{1}$$

We define the supermolecular ground state determinant Φ_0 as the reference vacuum and apply normal ordering:

$$\begin{aligned}
\hat{H}_N &= \hat{H}^{KS} - \langle \Phi_0 | H^{KS} | \Phi_0 \rangle \\
&= \sum_p \varepsilon_p \{a_p^\dagger a_p\} \\
&= \sum_a \varepsilon_a \{a_a^\dagger a_a\} + \sum_i \varepsilon_i \{a_i^\dagger a_i\} \\
&= \sum_a \varepsilon_a a_a^\dagger a_a - \sum_i \varepsilon_i a_i a_i^\dagger
\end{aligned} \tag{2}$$

The curly braces indicate normal ordering with respect to Φ_0, while indices i and a label occupied and unoccupied (virtual) supermolecular orbitals, respectively. With this definition the energy of singly excited supermolecular states like $\Phi_j^b = a_b^\dagger a_j |0\rangle$ is simply given by the orbital energy difference $\varepsilon_b - \varepsilon_j$.

Before introducing the non-orthogonal fragment orbitals it is convenient to redefine the original creation and annihilation operators in terms of hole/particle operators as $b_i = a_i^\dagger$, $b_i^\dagger = a_i$, $b_a = a_a$ and $b_a^\dagger = a_a^\dagger$. We also redefine the orbital energies accordingly: $\epsilon_i = -\varepsilon_i$, $\epsilon_a = \varepsilon_a$. The double summation defined above then reduces again to a single summation

over all supermolecular orbitals. We then consider building locally excited states and charge transfer states that are each defined as single excitations on the original (supermolecular) vacuum. For this we use a set of fragment orbitals that spans the same space as the supermolecular basis (and is typically, but not necessarily, expressed in the same underlying atomic orbital basis) but is partly non-orthogonal. We assume that the formation of the interacting supermolecule from the non-interacting fragments does not lead to strong mixing of the occupied and virtual spaces of the fragments so that their combined occupied space spans the supermolecular occupied space and their combined virtual space spans the supermolecular virtual space. We will label these fragment orbitals with the Greek indices κ_A, λ_A and α_A, β_A for occupied and virtual orbitals on fragment A, respectively, and by π_A, ρ_A when relating to all orbitals of fragment A. In this basis we have

$$[b^\dagger_{\pi_A}, b^\dagger_{\rho_B}]_+ = 0 \tag{3}$$

$$[b_{\pi_A}, b_{\rho_B}]_+ = 0 \tag{4}$$

$$[b^\dagger_{\pi_A}, b_{\rho_B}]_+ = S_{\rho_B,\pi_A} \tag{5}$$

with the overlap matrix elements defined as

$$S_{\rho_B,\pi_A} = \int \phi^*_{\rho_B}(\mathbf{x})\phi_{\pi_A}(\mathbf{x})d\mathbf{x} \tag{6}$$

We have partial orthogonality between either orbitals on the same fragment: $S_{\rho_A,\pi_A} = \delta_{\rho,\pi}$ as well as between occupied and virtual orbitals in general: $S_{\kappa_A\alpha_B} = 0$. The fragment orbitals are non-orthogonal to supermolecular orbitals of the same kind (occupied or virtual), i.e. $S'_{i,\kappa_A} \neq 0$ and $S'_{a,\alpha_A} \neq 0$ with

$$S'_{p,\pi_A} = \int \psi^*_p(\mathbf{x})\phi_{\pi_A}(\mathbf{x})d\mathbf{x}, \tag{7}$$

so that the anticommutation relations of their operators become

$$[b^{\dagger}_{\pi_A}, b^{\dagger}_{p}]_+ = 0 \tag{8}$$

$$[b_{\pi_A}, b_{p}]_+ = 0 \tag{9}$$

$$[b^{\dagger}_{\pi_A}, b_{p}]_+ = S'_{p,\pi_A}. \tag{10}$$

In the following we will temporarily suppress the label for the subsystem for ease of notation, keeping otherwise the same conventions. We are interested in the LE/CT couplings that can be expressed in the following general way

$$\begin{aligned}\langle\Phi^{\alpha}_{\kappa}|\hat{H}_N|\Phi^{\beta}_{\lambda}\rangle &= \sum_p \epsilon_p \langle 0|b_\kappa b_\alpha b^{\dagger}_p b_p b^{\dagger}_\beta b^{\dagger}_\lambda|0\rangle \\ &= \sum_p \epsilon_p (S_{\kappa\lambda}S'_{p\alpha}S'_{p\beta} - S_{\kappa\beta}S'_{p\lambda}S'_{p\alpha} - S_{\alpha\lambda}S'_{p\kappa}S'_{p\beta} + S_{\alpha\beta}S'_{p\kappa}S'_{p\lambda}) \\ &= \sum_p \epsilon_p (S_{\kappa\lambda}S'_{p\alpha}S'_{p\beta} + S_{\alpha\beta}S'_{p\kappa}S'_{p\lambda}),\end{aligned} \tag{11}$$

where we have used the fact that we work with real orbitals in the second line and the general orthogonality of the occupied and virtual orbitals in the third line. The desired LE/CT coupling elements are thus

$$V^{LE_A,CT_{A\to B}} = \langle\Phi^{\alpha_A}_{\kappa_A}|\hat{H}_N|\Phi^{\beta_B}_{\lambda_A}\rangle = \sum_a \epsilon_a \delta_{\kappa\lambda} S'_{a\alpha_A} S'_{a\beta_B} + \sum_i \epsilon_i S_{\alpha_A,\beta_B} S'_{i,\kappa_A} S'_{i,\lambda_A} \tag{12}$$

$$V^{LE_A,CT_{B\to A}} = \langle\Phi^{\alpha_A}_{\kappa_A}|\hat{H}_N|\Phi^{\beta_A}_{\lambda_B}\rangle = \sum_i \epsilon_i \delta_{\alpha\beta} S'_{i\kappa_A} S'_{i\lambda_B} + \sum_a \epsilon_a S_{\kappa_A,\lambda_B} S'_{a,\alpha_A} S'_{a,\beta_A}. \tag{13}$$

Since we consider charge-transfer to occur from the highest occupied molecular orbital of one fragment to the lowest unoccupied orbital of another fragment, these equations can be further simplified to:

$$V^{Q_y^A,CT_{A\to B}} = \sum_a \varepsilon_a S'_{a,L_A} S'_{a,L_B} - \sum_i \varepsilon_i S_{L_A,L_B} {S'}^2_{i,H_A} \quad (14)$$

$$V^{Q_y^A,CT_{B\to A}} = -\sum_i \varepsilon_i S'_{i,H_A} S'_{i,H_B} + \sum_a \varepsilon_a S_{H_A,H_B} {S'}^2_{a,L_A} \quad (15)$$

$$V^{Q_x^A,CT_{A\to B}} = -\sum_i \varepsilon_i S_{L_A,L_B} S'_{i,H'_A} S'_{i,H_A} \quad (16)$$

$$V^{Q_x^A,CT_{B\to A}} = -\sum_i \varepsilon_i S'_{i,H'_A} S'_{i,H_B} + \sum_a \varepsilon_a S_{H'_A,H_B} {S'}^2_{a,L_A} \quad (17)$$

in which the HOMO-1 of a fragment A that is responsible for the Q_x LE states is labeled as H'_A. We switched back to the original orbital energies in the above equations. In these four unique coupling elements we recognize the contributions of hole as well as electron transfer, which are respectively governed by the overlap between the occupied and the virtual orbitals of the individual fragments.

The overlaps between the different states are given as

$$\begin{aligned} \langle \Phi_\kappa^\alpha | \Phi_\lambda^\beta \rangle &= \langle 0 | b_\kappa b_\alpha b_\beta^\dagger b_\lambda^\dagger | 0 \rangle \\ &= (S_{\alpha\beta} S_{\kappa\lambda} - S_{\alpha\lambda} S_{\kappa\beta}) \\ &= S_{\alpha\beta} S_{\kappa\lambda} \end{aligned} \quad (18)$$

with the third line following from the assumption of orthogonality between the occupied and virtual spaces. Therefore the necessary overlaps between the considered states are,

$$S^{Q_y^A,CT_{A\to B}} = S_{L_A,L_B} \quad (19)$$

$$S^{Q_y^A,CT_{B\to A}} = S_{H_A,H_B} \quad (20)$$

$$S^{Q_x^A,CT_{A\to B}} = S_{L_A,L_B} S_{H'_A,H_A} = 0 \quad (21)$$

$$S^{Q_x^A,CT_{B\to A}} = S_{H'_A,H_B} \quad (22)$$

Equations 21 results from the orthogonality of the occupied space for each fragment.

Effect of environment on the Energies and Couplings

Table S1: The averaged LE and CT energies in vacuum (E_{VAC}) and in a DRF environment (E_{DRF}) from the first and the last set of frames of the trajectory. All energies are given in eV.

	First50		Last50	
	$\langle E_{VAC} \rangle$	$\langle E_{DRF} \rangle$	$\langle E_{VAC} \rangle$	$\langle E_{DRF} \rangle$
Q_{y1}	2.03	2.02	2.04	2.03
Q_{y2}	2.01	2.01	2.01	1.99
Q_{x1}	2.30	2.27	2.31	2.28
Q_{x2}	2.26	2.26	2.27	2.21
$CT1$	2.81	2.77	2.59	2.59
$CT2$	3.20	3.28	2.51	2.52

Table S2: The average absolute LE/LE and LE/CT couplings (eV) in vacuum ($|V_{@VAC}|$) and in DRF environment ($|V_{@DRF}|$) from the first and the last set of frames of the trajectory. See main text for the definition of these couplings.

	First50		Last50	
	$\langle |V_{@VAC}| \rangle$	$\langle |V_{@DRF}| \rangle$	$\langle |V_{@VAC}| \rangle$	$\langle |V_{@DRF}| \rangle$
V_{Qy}	0.018	0.021	0.020	0.029
V_{Qx}	0.007	0.009	0.011	0.017
$V_{Q_yA/CT1}$	0.009	0.009	0.014	0.014
$V_{Q_yA/CT2}$	0.017	0.019	0.066	0.054
$V_{Q_xA/CT2}$	0.017	0.017	0.059	0.062
$V_{Q_yB/CT1}$	0.017	0.019	0.067	0.055
$V_{Q_yB/CT2}$	0.008	0.009	0.014	0.014
$V_{Q_xB/CT1}$	0.017	0.015	0.121	0.128

Effect of environment on Transition Dipoles

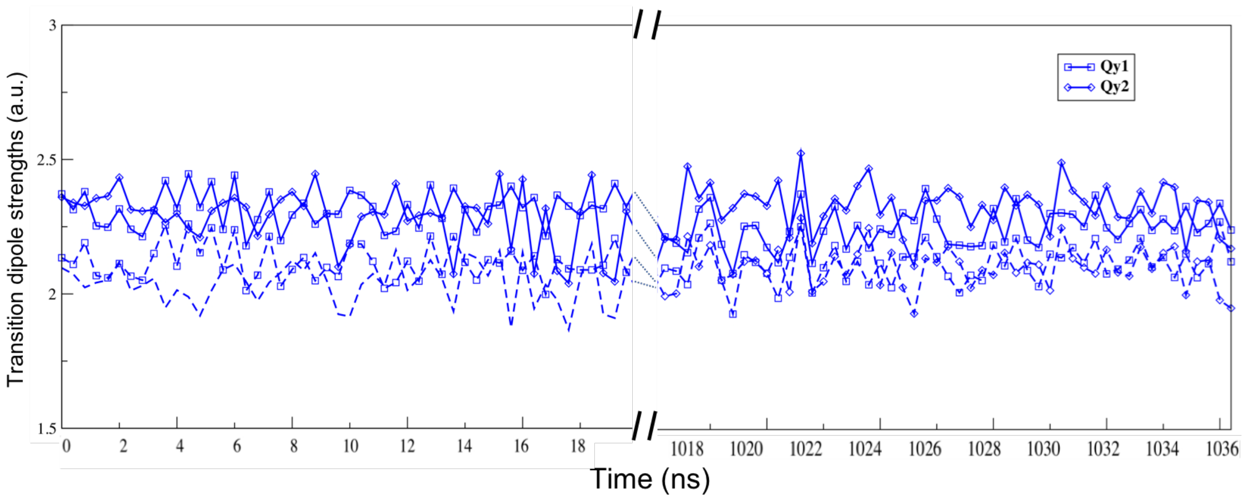

Figure S1: Absolute values of transition dipole moments for Q_{y1} and Q_{y2} in vacuum (denoted by dotted lines) and in DRF environment (denoted by bold lines) from the beginning towards the end (left and right of the split on x-axis) of the trajectory are shown. Dipole moments are in a.u.
